# Porcupine‐Quill‐Inspired Shape‐Morphing Biointerfaces Disassemble Biofilms

**DOI:** 10.1002/advs.78251

**Published:** 2026-10-12

**Authors:** Feng Qian, Muqiu Xia, Ziyu Wang, Xinyuan Jia, Jinke Yin, Mengyu Fu, Xue Zhang, Xiao‐Lei Shi, Shiqiang Chen, Ning Wang, Zhi‐Gang Chen

**Affiliations:** ^1^ Institute of Marine Science and Technology Shandong University Qingdao Shandong China; ^2^ Shandong Key Laboratory of Intelligent Marine Engineering Geology Environment and Equipment Institute of Marine Science and Technology Shandong University Qingdao Shandong China; ^3^ ARC Research Hub in Zero‐emission Power Generation for Carbon Neutrality Centre for Materials Science and School of Chemistry and Physics Queensland University of Technology Brisbane Queensland Australia; ^4^ College of Chemical Engineering Qingdao University of Science and Technology Qingdao Shandong China

**Keywords:** biofilm disassembly, mechano‐bactericidal surface, nano‐dagger array, offensive bio‐interface, surface chemistry

## Abstract

Mature biofilms are difficult to eradicate because the extracellular polymeric substance (EPS) matrix shields embedded bacteria from chemical and mechanical attack. Here, we report a porcupine‐spine‐inspired photo‐responsive dynamic offensive bio‐interface that dismantles mature biofilms through electrically actuated mechanical penetration and light‐driven oxidative stress. A high‐aspect‐ratio nano‐dagger array undergoes reversible nanoscale deformation (∼110 nm) under an alternating electric field, while a photo‐responsive heterostructure generates a surface photovoltage (∼364 mV) that enhances reactive oxygen species production. Mechanical penetration continuously disrupts the biofilm–surface interface, exposing bacteria within the EPS matrix to oxidative attack. This synergistic mechanism enables nearly complete biofilm eradication under complete immersion in bacterial suspensions of 10^9^ CFU mL^−1^, outperforming representative state‐of‐the‐art systems while maintaining stable antibiofilm activity for at least 10 days without chemical biocides. This work establishes a dynamic offensive strategy for durable self‐cleaning and antifouling interfaces.

## Graphical abstract

A porcupine‐spine‐inspired dynamic offensive bio‐interface is developed for active biofilm disassembly. By coupling electrically driven nano‐dagger penetration with light‐triggered ROS generation, the surface overcomes the passive limitations of conventional mechano‐bactericidal topographies, achieving near‐complete biofilm eradication, strong mechanical disruption, and durable self‐cleaning antibiofilm activity.

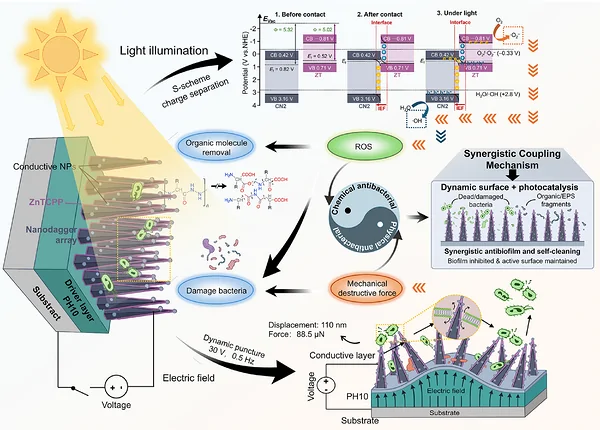

## 1 Introduction

Biofilm formation on solid interfaces remains a major challenge in marine, biomedical, and industrial environments because it causes persistent contamination, accelerates interfacial degradation, and compromises the long‐term reliability of functional materials [1, 2, 3]. In particular, biofilms are highly organized multicellular communities embedded in a self‐produced EPS matrix, which reinforces adhesion, preserves structural integrity, and protects resident microorganisms from environmental stresses and antimicrobial agents [4, 5, 6, 7]. As a result, mature biofilms are far more difficult to eradicate than planktonic cells, and effective control requires not only prevention of initial attachment but also active disruption of the biofilm‐surface interface before irreversible deterioration occurs [8, 9].

Current antibiofilm strategies mainly rely on anti‐adhesion coatings, biocide‐releasing systems, and mechano‐bactericidal (MB) nano‐topographies [10, 11, 12, 13]. However, these approaches remain limited. Release‐based coatings often suffer from burst release and declining long‐term efficacy as surviving microorganisms develop enhanced tolerance [14, 15, 16, 17]. Static topographical surfaces are typically effective only at early colonization stages and often provide insufficient destructive potency to dismantle EPS‐rich mature biofilms. Their micro/nanostructures can also be progressively passivated by accumulated biomass and organic foulants, leading to time‐dependent loss of activity [18, 19, 20, 21, 22]. Although light‐responsive oxidative strategies offer chemical‐free microbial inactivation, their performance against mature biofilms is restricted by limited ROS penetration and the protective biofilm microenvironment [23, 24, 25]. Recent studies have highlighted the importance of integrating nanostructured architectures, interfacial regulation, and stimulus‐responsive functions to develop advanced antibiofilm materials with enhanced activity and durability [26, 27, 28]. These developments provide important context for the construction of the dynamic photo‐responsive nano‐dagger interface proposed in this work. Therefore, an unresolved challenge is to develop an antibiofilm interface that can simultaneously deliver strong mechanical disruption, efficient ROS generation, and sustained operational stability.

Inspired by the offensive defense strategy of porcupine quills, we developed a photo‐responsive dynamic offensive bio‐interface that couples electrically driven mechanical penetration with light‐triggered oxidative stress. A high‐aspect‐ratio tetrakis (4‐carboxyphenyl) zinc porphyrin (ZT)‐loaded NiCo(OH)_2_CO_3_ (ZTCN2) nano‐dagger array was integrated onto an HfO_2_‐modified polydimethylsiloxane (PDMS) driving layer (PH*x*, where *x* denotes the HfO_2_/PDMS w/v ratio), enabling reversible electromechanical actuation together with enhanced photo‐responsive activity. Here, CN*y* represents NiCo(OH)_2_CO_3_ with different Co/Ni ratios, where *y* is the Co‐to‐Ni ratio. This hierarchically ordered architecture transforms conventional passive antibiofilm surfaces into an active interface capable of both disrupting mature biofilms and maintaining long‐term activity through continuous exposure of active features and removal of residual biomass. Under coupled light‐electric stimulation, the resulting surface achieves nearly 100% antibiofilm efficiency while maintaining stable operation for 10 days. Overall, this work establishes a dynamic antibiofilm design strategy for durable self‐cleaning and biofilm‐control interfaces.

## 2 Results and Discussion

### 2.1 Porcupine‐Spine‐Inspired Design of a Dynamic Offensive Antibiofilm Interface

To overcome the limitations of conventional MB surfaces, we drew inspiration from the offensive defense strategy of porcupine quills. Unlike static MB surfaces that mainly rely on passive antibacterial action, erect porcupine quills act as active piercing weapons (Figure 1a,b). Inspired by this structure‐function relationship, we designed a bioinspired antibacterial interface featuring nano‐dagger‐like protrusions and a photo‐responsive functional layer, enabling dynamic mechanical penetration together with light‐triggered oxidative damage and thereby transforming passive interfacial protection into active disassembly of mature biofilms (Figure 1c).

**FIGURE 1:**
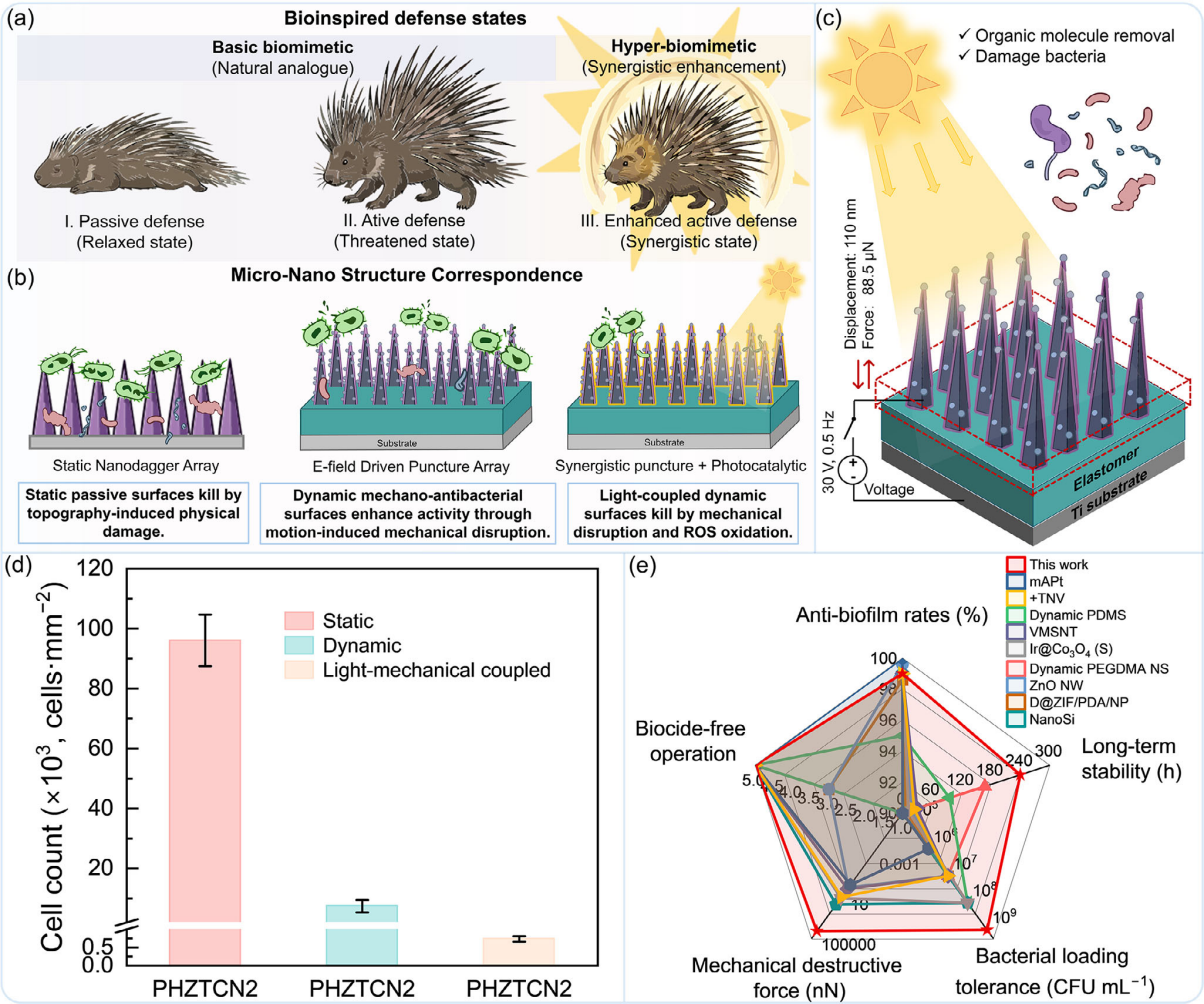
Bioinspired design of the light‐responsive dynamic micro/nanostructured interface. (a, b) Schematic illustrations of the micro/nanostructure inspired by the defensive mechanism of porcupine quills. (c) Schematic of the hierarchically integrated light‐responsive dynamic micro/nanostructured interface. (d) The cell count results of surfaces prepared according to the concept under static, dynamic, and light‐mechanical synergy. (e) Radar‐chart comparison of the overall antibiofilm performance of the present work with representative antibiofilm surfaces reported in the literature, including core‐shell mesoporous Au@Pt (mAPt [29]), charged vertical titanium nitride (+TNV [30]), dynamic PDMS [23], virus‐like mechano‐bactericidal nanomotor (VMSNT [31]), spiky Ir@Co_3_O_4_ particles (Ir@Co_3_O_4_ (S) [20]), dynamic poly(ethylene glycol) dimethacrylate nanosheets (dynamic PEGDMA NS [21]), ZnO nanowires (ZnO NW [22]), 3,3’‐diaminodipropylamine/ZIF‐8/polydopamine nanopillar (D@ZIF/PDA/NP [32]) and etching of silicon (NanoSi [33]). Comparison metrics include antibiofilm rate (%), long‐term stability (h), bacterial loading tolerance (CFU mL^−1^), Mechanical destructive force (nN), and Biocide‐free operation (Biocide‐release dependence was ranked from 1 to 5, where 5 represents fully biocide‐free operation and 1 represents a predominantly release‐based antibacterial mechanism). CFU, colony‐forming units; h, hours; NW, nanowires; NS, nanosheets.

The proposed bio‐interface consists of an HfO_2_‐modified PDMS dielectric elastomer actuator and a ZTCN2 photoactive nano‐dagger layer. Under electrical stimulation, the actuator drives periodic vertical motion of the nano‐dagger array to mechanically disrupt bacterial membranes and biofilm architecture, while light irradiation triggers ROS generation to further amplify bacterial damage and degrade accumulated foulants. Unlike conventional passive MB surfaces, this design integrates dynamic mechanical attack with photo‐induced self‐cleaning, helping preserve long‐term antibacterial activity.

Following this design rationale, the antibiofilm performance of PHZTCN2 was evaluated under static, dynamic, and light‐mechanical coupled conditions. The same surface exhibited markedly different antibiofilm behaviors under the three modes. Static conditions retained the highest bacterial count, indicating that the interface alone was insufficient to suppress biofilm accumulation. Dynamic actuation significantly reduced bacterial retention, whereas light‐mechanical coupling produced the lowest bacterial count, confirming the strongest antibiofilm effect through the synergistic combination of dynamic penetration and photo‐responsive ROS generation (Figure 1d).

The performance of MB surfaces is inherently context dependent, direct comparison based only on endpoint antibiofilm efficiency can be misleading. A meaningful evaluation must also consider bacterial loading, treatment conditions, resistance to passivation, and long‐term operational stability. In this context, the proposed bio‐interface is best assessed by its integrated performance. Compared with representative state‐of‐the‐art MB surfaces, it combines near‐complete biofilm eradication, high bacterial‐loading tolerance, prolonged operational stability, strong mechanical disruption, and biocide‐free operation. Importantly, it achieves nearly complete eradication of mature biofilms under complete immersion in bacterial suspensions containing 10^9^ CFU mL^−1^, an order of magnitude higher than the bacterial concentrations typically employed in previous studies (∼10^8^ CFU mL^−1^). In addition, the interface maintains stable antibiofilm performance for at least 10 days, extending the longest representative operational lifetime reported previously (∼7 days) by approximately 43%. Moreover, the interface delivers a penetration force of 88.5 µN, approximately 440 fold greater than the highest value reported for representative static or weakly dynamic mechanically bactericidal surfaces (∼200 nN). Together, these features enable rapid biofilm disassembly, sustained self‐cleaning, and durable antibiofilm protection (Figure 1e).

### 2.2 Construction of the Layered Nano‐Dagger Architecture and Its Functional Components

Following the establishment of the design concept, the hierarchical nano‐dagger architecture and its integrated functional modules were systematically characterized. Because the proposed offensive mechanism relies on the synergistic integration of a dielectric elastomer actuator, a high‐aspect‐ratio micro/nanostructure, and photo‐responsive active components, comprehensive characterization of the morphology, hierarchical architecture, and chemical composition was essential. Scanning electron microscopy (SEM) confirmed the successful formation of the designed hierarchical nano‐dagger array, while spectroscopic analyses verified the incorporation of the individual functional components into the composite interface. Together, these results establish the structural and compositional foundation underpinning the electrically driven actuation and light‐responsive antibiofilm functions demonstrated in the following sections.

The upper micro/nanostructured layer was approximately 22 µm thick and consisted of hydrothermally grown CN*y* arrays. SEM images showed that the resulting high‐aspect‐ratio nanostructures closely resembled porcupine quills, forming a biomimetic nano‐dagger architecture. The Co/Ni ratio markedly influenced the morphology, density, and dimensions of the arrays (Figure S1). Among the investigated compositions, CN2 exhibited the sharpest tips (≈50 nm) and the highest array density (≈6 × 10^6^ mm^−2^), both favorable for bacterial membrane penetration (Figures S2–S5). By contrast, increasing the Co/Ni ratio to 8 completely suppressed the nano‐dagger morphology (Figure S6), indicating that the Co/Ni ratio regulates nucleation and anisotropic growth. To probe the effect of morphology on antibacterial performance, Ti, CN2, CN4, and CN8 were immersed in *P. aeruginosa*‐containing seawater under static conditions and analyzed by laser scanning microscopy (CLSM). CN2 showed the strongest dead‐cell fluorescence signal, with an antibiofilm efficiency of ∼10.2% and a bactericidal rate of 7.5% (Figures S7 and Figure S8), outperforming the other surfaces. These results indicate that sharper and denser nano‐dagger arrays enhance physical disruption of bacterial cells and improve antibacterial activity.

To introduce photo‐responsive functionality, ZT was deposited onto CN2 to form the ZTCN2 composite. The UV‐vis absorption spectra of free‐based TCPP and the synthesized ZT were further recorded in the 500–750 nm region (Figure S9). Free‐based TCPP exhibited four Q‐band absorption features, whereas ZT displayed two dominant Q‐band peaks with a concomitant red shift. The reduction in the number of Q bands and the red‐shifted absorption are characteristic of porphyrin metalation, resulting from the increased molecular symmetry and modified electronic structure following Zn^2+^ coordination. Pristine ZT displayed a spindle‐like morphology (Figure S10), whereas ZT deposition caused no obvious change in the morphology or dimensions of the nano‐dagger array, indicating formation of a conformal amorphous coating on CN2 (Figure 2a). Transmission electron microscopy (TEM) and high‐resolution TEM further revealed lattice fringes with a spacing of 0.254 nm, corresponding to the (012) plane of CN2, while the ZT layer remained amorphous, consistent with the preserved nano‐dagger morphology after modification (Figure 2b,c). Together with the X‐ray diffraction (XRD) results, these observations confirm the good crystallinity of the ZTCN2 composite (Figure S11a) [34, 35]. Cross‐sectional SEM revealed that the photo‐responsive dynamic MB interface possessed a well‐defined hierarchical architecture (Figure 2d). Successful incorporation of ZT was additionally verified by attenuated total reflection Fourier‐transform infrared (ATR‐FTIR) and Raman spectroscopy. Compared with CN2, ZTCN2 exhibited additional vibrational bands associated with the porphyrin framework, confirming successful integration of the ZT component into the composite (Figures S11b,c) [36, 37]. Collectively, these results confirm successful fabrication of the ZTCN2 photo‐responsive nano‐dagger architecture.

**FIGURE 2:**
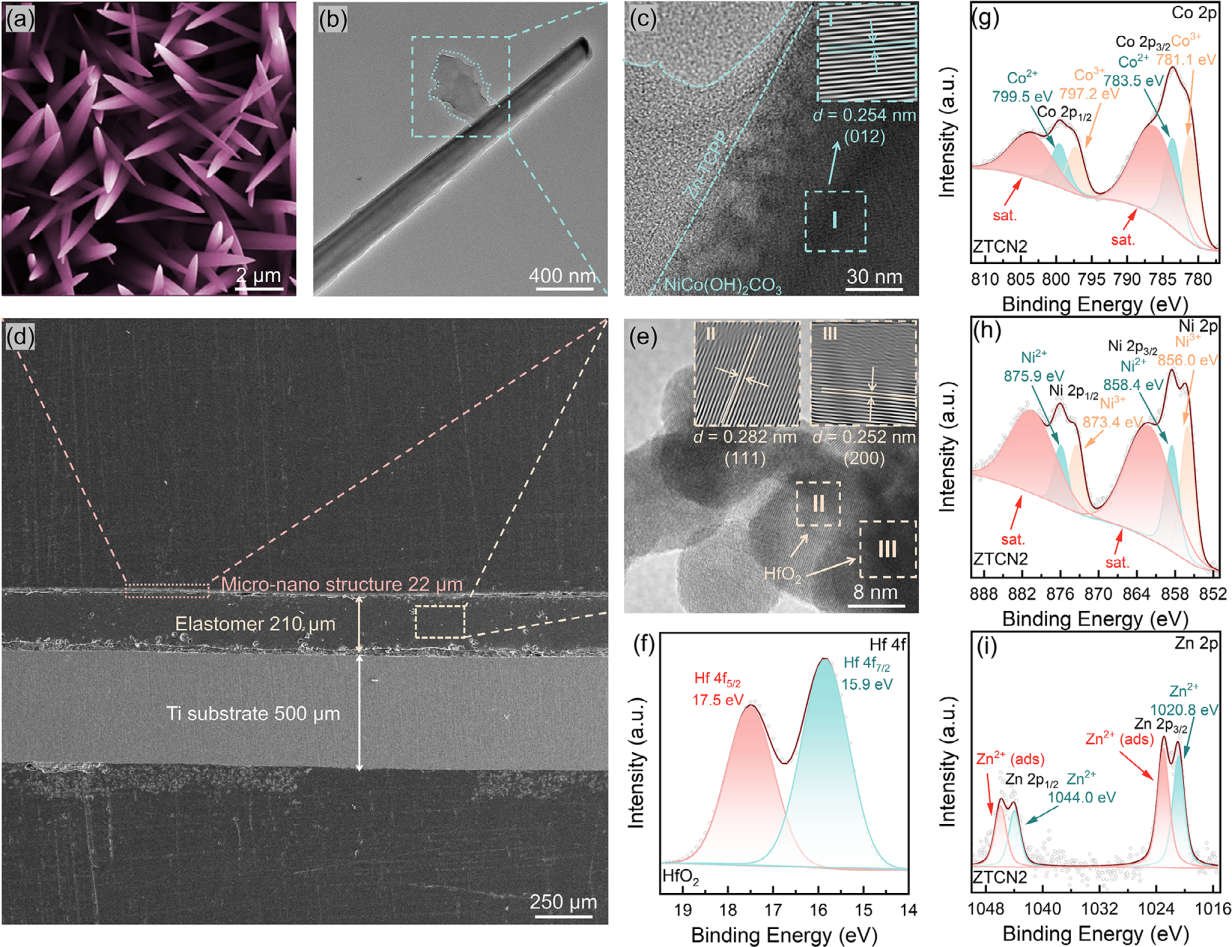
Characterization of the layered integrated components of PHZTCN2. (a) Scanning electron microscopy (SEM) image of ZTCN2. (b, c) Transmission electron microscopy (TEM) images of ZTCN2. (d) Cross‐sectional SEM image of the hierarchically integrated interface. (e) TEM image and (f) high‐resolution X‐ray photoelectron spectroscopy (XPS) Hf 4*f* spectrum of HfO_2_. High‐resolution XPS spectra of (g) Co 2*p*, (h) Ni 2*p*, and (i) Zn 2*p* for ZTCN2.

Beneath the micro/nanostructured layer is the PH*x* dielectric elastomer actuation layer with a thickness of approximately 210 µm, followed by a protected Ti substrate approximately 500 µm thick. HfO_2_ nanoparticles were introduced to provide the dielectric enhancement required for electrically driven deformation under low‐voltage stimulation. To verify the structure and composition of HfO_2_, its morphology, crystal structure, and chemical states were characterized by TEM, XRD, and X‐ray photoelectron spectroscopy (XPS). These analyses also showed that a loading of 10 wt/v% provided the best balance between dielectric and mechanical performance. TEM revealed uniformly dispersed HfO_2_ nanoparticles with diameters of ∼15–20 nm (Figure S12a). High‐resolution TEM showed lattice fringes with spacings of 0.282 and 0.252 nm, corresponding to the (111) and (200) planes of HfO_2_ (Figure 2e). XRD confirmed the high crystallinity of HfO_2_, with diffraction peaks matching the standard PDF#78‐0050 (Figure S12b) [38, 39, 40]. Raman spectroscopy provided complementary evidence for crystalline HfO_2_ (Figure S12c) [41]. XPS further verified HfO_2_ formation, showing characteristic Hf^4+^ doublets and lattice/surface oxygen contributions (Figure 2f and Figure S13) [38, 42]. Together, these results demonstrate successful synthesis of highly crystalline HfO_2_ nanoparticles suitable for dielectric enhancement of PDMS.

The chemical states of ZTCN2 were further investigated by XPS. The Co 2*p* and Ni 2*p* spectra displayed characteristic Co^3+^/Co^2+^ and Ni^3+^/Ni^2+^ doublets together with satellite peaks, confirming the mixed‐valence nature of the Ni‐Co hydroxide carbonate framework [43, 44]. The Zn 2*p* spectrum could be deconvoluted into Zn 2*p*_3/2_ and Zn 2*p*_1/2_ components, indicating the presence of Zn^2+^ species together with surface‐adsorbed Zn^2+^ (Figure 2g–i) [45]. In addition, the C 1*s* spectrum contained C─C, C═N/C─O, O─C═O, and π–π* components, while the O 1*s* spectrum showed contributions from hydroxyl groups, carbonate species, and adsorbed water, further supporting the carbonate‐containing framework (Figure S14a,b) [46, 47].

To optimize the electromechanical performance of the dielectric elastomer actuator, HfO_2_ nanoparticles were incorporated into PDMS at different mass/volume ratios, and the dielectric, electrical, and mechanical properties were systematically evaluated. Compared with pristine PDMS, the dielectric constant initially increased and then decreased with increasing HfO_2_ loading, reaching a maximum value of 4.9 at low frequency for PH10 (Figure S15a). PH10 also exhibited the best balance between dielectric enhancement and dielectric‐loss suppression, whereas higher HfO_2_ loading increased dielectric loss, likely because of nanoparticle aggregation and leakage pathways (Figure S15b) [48]. All samples maintained low electrical conductivity (<10^−6^ S cm^−1^) in the low‐frequency range (Figure S15c). Mechanical testing further showed that PH10 had the highest elastic modulus (∼680 kPa), providing a favorable balance between compliance and actuation capability (Figure S16a,b). These results identify PH10 as the optimal dielectric elastomer actuator. Integration of the photo‐responsive ZTCN2 nano‐dagger array onto PH10 then yielded the hierarchically integrated PHZTCN2 interface, which serves as the platform for the dynamic offensive antibiofilm strategy described below.

### 2.3 Photo‐Responsive Enhancement and Electrically Driven Actuation Enable Dual‐Mode Functionality

The next step was to verify whether the engineered interface possessed the two functions required for active biofilm disassembly: enhanced photo‐responsive activity and electrically driven dynamic actuation. Because oxidative attack depends on effective photoinduced charge separation, whereas mechanical disruption requires controllable interfacial deformation, both functions were examined before antibiofilm testing. Surface‐potential and photoelectrochemical analyses showed that the heterostructured active layer promoted charge separation under illumination, providing the basis for enhanced ROS generation. Meanwhile, the composite interface exhibited reversible deformation under an applied electric field, confirming dynamic actuation of the nano‐dagger architecture. Together, these results establish the functional basis for the synergistic coupling of mechanical penetration and light‐triggered oxidative attack.

The photo‐responsive properties of the fabricated surfaces were first evaluated by ultraviolet‐visible diffuse reflectance spectroscopy (UV‐vis DRS). CN2, CN4, and CN8 exhibited similar absorption edges at approximately 392 nm, whereas incorporation of ZT into CN2 produced a pronounced red shift to 641 nm, extending the light‐response range of ZTCN2 into the visible region (Figure S17). Kelvin probe force microscopy (KPFM) further revealed a substantial enhancement in the photoinduced surface potential after ZT modification (Figure 3a,b). Under dark conditions, CN2 and ZTCN2 exhibited similar surface potentials of ∼60 mV. Upon illumination, however, the surface potential of ZTCN2 increased to ∼364 mV, more than three times that of CN2 (112 mV), indicating markedly improved charge separation (Figure 3c,d). The electrochemically active surface area (ECSA) was then evaluated by cyclic voltammetry (CV), and the corresponding double‐layer capacitance (*C*_dl_) and ECSA values were determined according to Equations (1) and (2) (Figure S18a–d) [49, 50, 51].

(1) $$C_{\mathrm{dl}}=\frac{I_{C}}{v}$$

(2) $$\mathrm{ECSA}\;=\frac{C_{\mathrm{dl}}}{C_{s}}$$

here, *I*_c_ represents the charging current at different scan rates, v is the scan rate, and Cs is the specific capacitance of the electrolyte. Because Cs remains constant for a given electrolyte, ECSA can be estimated from *C*_dl_. CN2, CN4, and CN8 showed comparable ECSA values, with CN2 only slightly higher. In contrast, ZT modification markedly increased the ECSA of ZTCN2, giving a *C*_dl_ of 250.6 µF cm^−2^ (Figure 3e and Figure S18e). This increase suggests a larger electrochemically accessible surface, which should facilitate interfacial charge transfer and photoinduced redox processes.

**FIGURE 3:**
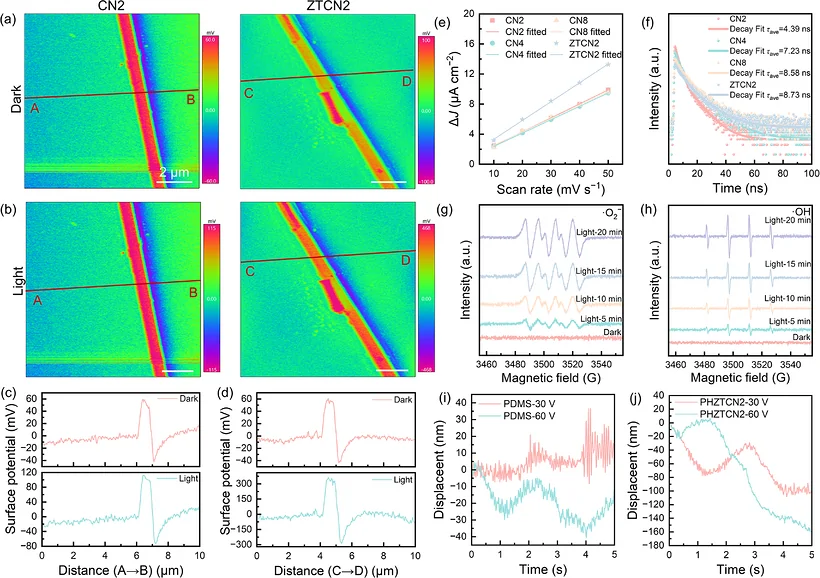
Characterization of the interfacial photoelectric response, charge‐transfer behavior, and electrically actuated deformation performance. (a, b) Kelvin probe force microscopy (KPFM) images and (c, d) surface‐potential maps of CN2 and ZTCN2 under dark and illuminated conditions. (e) Electrochemically active surface area (ECSA) of CN2, CN4, CN8, and ZTCN2. (f) Time‐resolved photoluminescence (TRPL) spectra of CN2, CN4, CN8, and ZTCN2 (*τ*_ave_ stands for average carrier lifetime). (g, h) Electron spin resonance (ESR) spectra of CN2 and ZTCN2. (i) Electrically driven deformation displacement of PDMS‐based dielectric elastomers under different applied voltages. (j) Reversible deformation displacement of the PHZTCN2 interface under different driving voltages.

Photoluminescence (PL) spectroscopy was used to evaluate recombination of photoinduced electron‐hole pairs. As the Co/Ni ratio increased, the PL intensity also increased, indicating accelerated charge‐carrier recombination. Among the CNy samples, CN2 exhibited the weakest PL emission, suggesting more efficient charge separation (Figure S19). After ZT modification, the PL intensity decreased further, indicating that the heterostructure effectively suppressed electron‐hole recombination. Time‐resolved photoluminescence (TRPL) measurements further showed that ZTCN2 had the longest average carrier lifetime (8.73 ns), demonstrating prolonged charge separation and improved utilization of photogenerated carriers (Figure 3f). Photo‐induced ROS generation was then probed by electron spin resonance (ESR) spectroscopy. No detectable ·OH or ·O_2_^−^ signals were observed under dark conditions, whereas both radical signals appeared under illumination and increased with irradiation time, confirming efficient ROS generation on the PHZTCN2 interface (Figure 3g,h). These results demonstrate that the photo‐responsive heterostructure effectively promotes charge separation and ROS production, providing the oxidative basis for the subsequent light‐enhanced antibiofilm performance.

These results demonstrate that PHZTCN2 exhibits enhanced photo‐responsive activity and efficient ROS generation, which together provide the oxidative basis for its antibiofilm function. This improvement arises from the heterostructure between ZT and CN2, which facilitates charge transfer and suppresses electron‐hole recombination. To verify the second key function, namely active mechanical penetration, the electrically driven deformation of pristine PDMS and PHZTCN2 was quantified using a high‐voltage driving module coupled with a laser displacement sensor. For both samples, deformation increased with voltage, but PHZTCN2 consistently exhibited much larger displacement than pristine PDMS. At 30 V, pristine PDMS showed a maximum displacement of only ∼36 nm, whereas PHZTCN2 achieved a reversible deformation of ∼110 nm (Figure 3i,j). Higher voltages produced larger deformation amplitudes and more pronounced periodic actuation. To ensure practical electrical safety, the operating voltage was kept below 36 V throughout this work (Figure S20). The enhanced electromechanical response of PHZTCN2 is attributed to the increased dielectric constant and improved electromechanical coupling resulting from HfO_2_ incorporation. After establishing the deformation behavior, the force exerted by PHZTCN2 on *P. aeruginosa* was estimated using the small‐deformation Maxwell stress model (Equation 3) [52, 53].

(3) $$F=\varepsilon_{0}\;\varepsilon_{r}\frac{V^{2}A_{0}}{d^{2}}$$

here, *ε*_0_ denotes the vacuum permittivity (8.85 × 10^−12^ F·m^−1^), *ε*_r_ is the dielectric constant of the elastomer, *V* is the applied voltage, *A*_0_ is the surface area, and *d* is the elastomer thickness. Using the experimentally determined parameters (*ε*_r_ = 4.9, *A*_0_ = 1 cm^2^, *d* = 210 µm, and *V* = 30 V), the maximum mechanical force generated by the dynamic interface was estimated to be 88.5 µN. The calculated force represents the total actuation force over the entire active area. Previous studies have reported that rupture of a bacterial cell membrane typically requires a force of only 7–25 nN [54]. Thus, the force generated by PHZTCN2 exceeds the reported membrane‐rupture threshold by several orders of magnitude, indicating that the dynamic nano‐dagger interface can mechanically penetrate and disrupt surface‐adhered *P. aeruginosa* cells. This strong electromechanical output provides the mechanical basis for the dynamic offensive antibiofilm mechanism proposed here.

Instantaneous electrical‐power measurements further showed that, at 30 V, PHZTCN2 exhibited a maximum positive instantaneous power of only ∼210 nW during a driving cycle, which was ∼41.7% lower than that of pristine PDMS (Figure S21). This result reflects the peak cycle‐resolved electrical response rather than the total energy consumption over an extended operating period. This improvement is attributed to reduced dielectric loss after HfO_2_ incorporation, which enhances the electromechanical efficiency of the dielectric elastomer. The combination of large reversible deformation and ultralow power consumption highlights the potential of PHZTCN2 as an energy‐efficient platform for long‐term dynamic antibiofilm applications.

### 2.4 Coupled Light‐Mechanical Treatment Achieves Efficient and Durable Antibiofilm Performance

Having established the photo‐responsive and electromechanical functionalities of the interface, we next examined whether coupling light stimulation with dynamic mechanical actuation could further enhance antibiofilm performance. The rationale was that dynamic penetration would disrupt biofilm integrity and improve interfacial accessibility, thereby facilitating ROS penetration and amplifying oxidative antibacterial activity under illumination. CLSM, quantitative fluorescence analysis, and bacterial viability assays consistently showed that the light‐mechanical coupled mode outperformed the static, light‐only, and mechanically driven modes in both suppressing biofilm formation and inactivating surface‐adhered bacteria. The interface also maintained high antibiofilm activity during long‐term testing. Together, these results identify synergistic light‐mechanical coupling as the dominant operating mechanism of the dynamic offensive bio‐interface.

The antibiofilm performance of PDMS, PH10, PHCN2, and PHZTCN2 was first evaluated under electrically driven actuation at 5, 15, and 30 V. Compared with static conditions, all actuated interfaces showed improved antibiofilm performance, with efficacy increasing progressively with voltage. This voltage dependence indicates that electrically driven actuation enhances antibiofilm activity by enabling active mechanical penetration. CLSM further showed that live‐bacteria fluorescence decreased whereas dead‐bacteria fluorescence increased with increasing voltage for all samples. Under identical conditions, surfaces incorporating nano‐dagger arrays consistently showed weaker live‐cell fluorescence and stronger dead‐cell fluorescence than the flat PDMS and PH10 controls (Figure 4a and Figure S22a,b), indicating more effective disruption of surface‐associated biofilms. Quantitative analysis showed that, at 30 V, PHZTCN2 achieved an antibiofilm efficiency of ∼91.5% and a bacterial killing efficiency of 43.4%, corresponding to 1.9 fold and 10.1 fold increases, respectively, relative to 5 V (Figure 4b,c and Figure S22c–e). This enhancement arises from reversible displacement of the nano‐dagger arrays, which enables repeated mechanical penetration and continuous disruption of biofilm architecture. Unlike passive nano‐topographies, electrically driven actuation converts the interface into an active offensive platform that persistently attacks bacterial cells and suppresses biofilm establishment.

**FIGURE 4:**
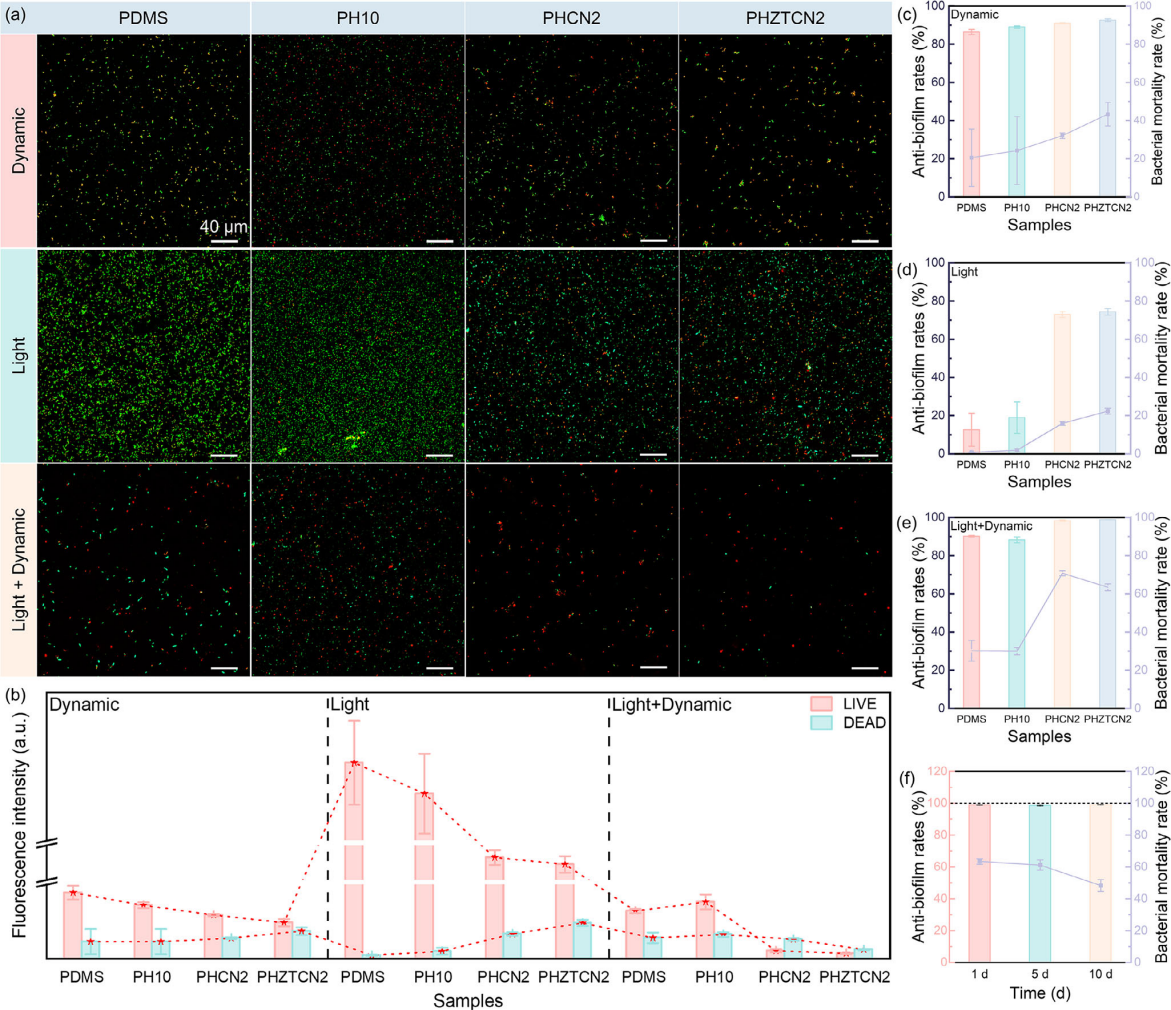
Characterization of the antibiofilm performance and long‐term operational stability of the light‐responsive dynamic micro/nanostructured interface. (a) Confocal laser scanning microscopy (CLSM) images of PDMS, PH10, PHCN2, and PHZTCN2 under mechanical, light, and light‐mechanical coupling conditions. (b) Quantitative fluorescence analysis of the CLSM images. (c‐e) Antibiofilm efficiency and bacterial killing efficiency of the different surfaces under the corresponding operating conditions. (f) Long‐term operational stability of the light‐responsive dynamic micro/nanostructured interface. Data are presented as the mean ± standard deviation (SD) (*n* = 3).

To further clarify the contribution of photoactivation, the antibiofilm performance of PHZTCN2 was evaluated under light‐only and light‐mechanical coupled conditions. To minimize heat accumulation during illumination, circulating cooling water was continuously applied throughout the antibiofilm experiments. Substantial bacterial survival remained in the light‐only group in the plate‐counting assay, indicating that irradiation alone under the cooled conditions exerted limited antibacterial activity. Therefore, photothermal heating was unlikely to be the dominant contributor to the observed antibiofilm performance, which was primarily attributed to the combined effects of photoinduced ROS generation and electrically driven mechanical perturbation. Minor local photothermal effects, however, cannot be completely excluded. CLSM and quantitative fluorescence analysis revealed that, under light irradiation alone, PDMS and PH10 exhibited only marginal antibiofilm activity, with antibiofilm efficiencies of 12.6% and 18.9%, respectively. Correspondingly, both surfaces displayed strong live‐bacteria fluorescence, reflecting the absence of photo‐responsive nano‐dagger structures (Figure 4d). In contrast, PHCN2 and PHZTCN2 showed pronounced antibiofilm activity owing to light‐induced ROS generation, with efficiencies approaching 70%. Notably, PHZTCN2 produced much stronger dead‐cell fluorescence than PHCN2, indicating that ZT modification enhances visible‐light utilization and photoinduced charge separation. Under simultaneous light and electromechanical stimulation, the antibiofilm performance of all interfaces improved markedly relative to light irradiation alone. Most notably, PHCN2 and PHZTCN2 achieved nearly complete biofilm eradication, with bacterial killing efficiencies of 63.4%–70.8%. These results demonstrate that dynamic penetration and photo‐induced ROS generation act synergistically to disrupt mature biofilm architecture, accelerate bacterial inactivation, and inhibit biofilm redevelopment. Accordingly, PHZTCN2 exhibited significantly better antibiofilm performance under light‐mechanical coupling than under either light‐only or mechanically driven conditions alone (Figure 4e), confirming that synergistic coupling is the dominant mechanism responsible for its exceptional activity.

The long‐term operational stability of PHZTCN2 was further evaluated under continuous biofilm‐forming conditions. Conventional MB surfaces are prone to passivation caused by accumulation of dead bacterial debris and EPS, which progressively compromises antibacterial performance. In contrast, the proposed light‐mechanical coupling strategy mitigates surface fouling through combined dynamic removal and photo‐induced degradation of organic residues. To assess durability, PHZTCN2 was continuously operated for 10 days while maintaining a constant bacterial concentration in the surrounding environment. Throughout the testing period, the interface retained nearly unchanged antibiofilm performance, demonstrating excellent operational stability and long‐term durability (Figure 4f and Figure S23). Moreover, it can be observed that after soaking for 6 days, the surface exhibits limited ion release (Figure S24). These results confirm that the dynamic offensive bio‐interface can sustain its antibiofilm functionality over extended operation without obvious performance degradation.

### 2.5 Dynamic Penetration and Oxidative Amplification Drive Active Biofilm Disassembly

To elucidate the origin of the exceptional antibiofilm performance of the coupled interface, the underlying disassembly mechanism was systematically investigated. We hypothesized that dynamic mechanical penetration by the nano‐dagger array would first compromise the integrity of bacterial envelopes and the EPS matrix, thereby facilitating deeper penetration of ROS and amplifying oxidative damage under light irradiation (Figure 5a,b). SEM revealed substantially greater disruption of the biofilm architecture under the light‐mechanical coupled mode than under either single‐mode treatment. Membrane‐permeability measurements and free‐bacteria assays further confirmed enhanced membrane damage and bacterial inactivation resulting from the coupled strategy. In addition, molecular dynamics (MD) simulations revealed stronger interfacial interactions between the nano‐dagger structures and bacterial membranes, providing molecular‐level evidence for penetration‐induced membrane disruption. Collectively, these results demonstrate that the superior antibiofilm performance of the dynamic offensive bio‐interface originates from the synergistic coupling of active mechanical penetration and amplified photo‐induced oxidative stress, which together drive efficient disassembly of mature biofilms.

**FIGURE 5:**
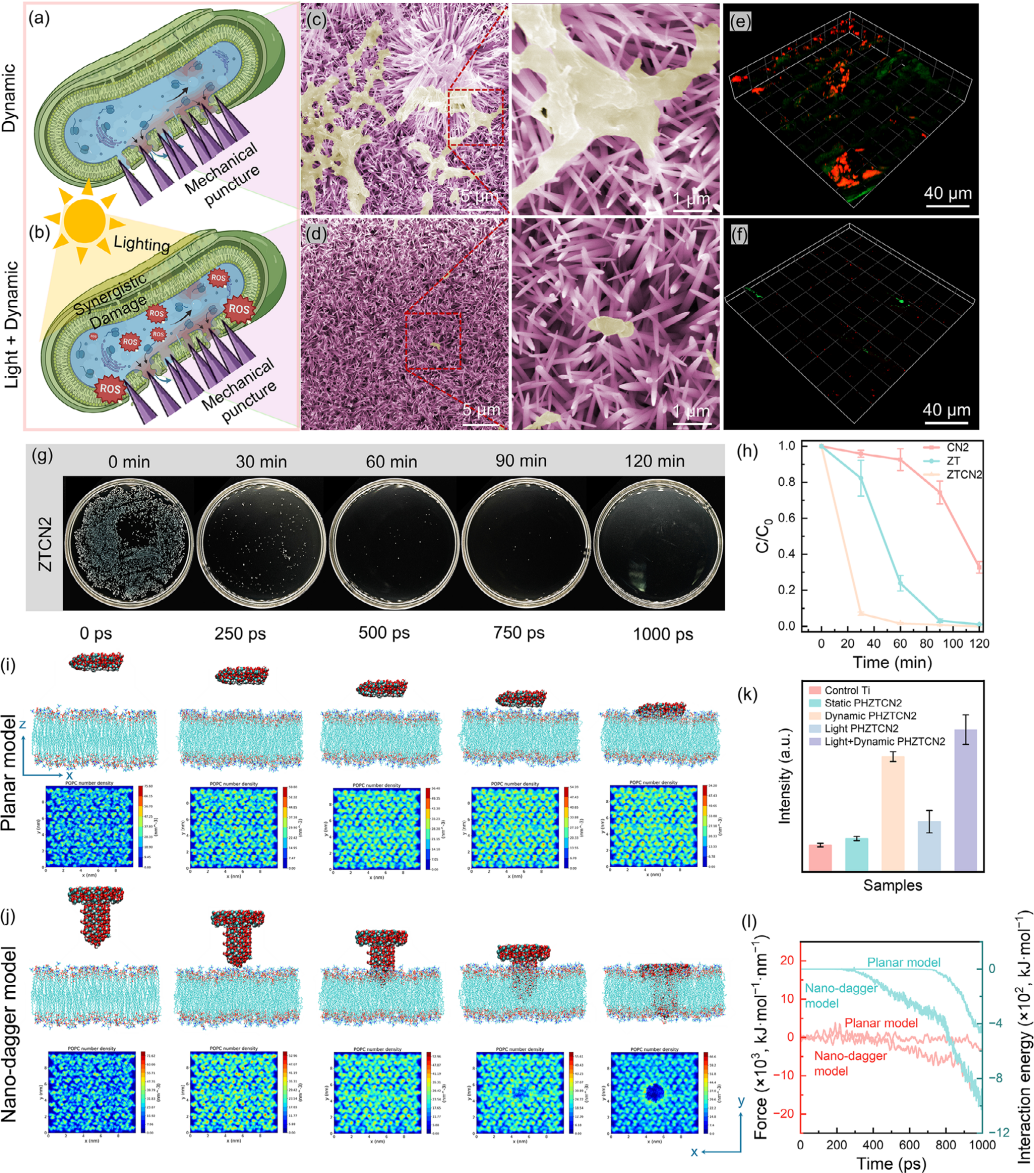
Proposed antibiofilm mechanism of the PHZTCN2 dynamic offensive bio‐interface. (a, b) Schematic illustration of the light‐mechanical coupled antibiofilm mechanism. (c, d) SEM images of PHZTCN2 after 1 day of incubation with *P. aeruginosa* under mechanically driven and light‐mechanical coupled conditions. (e, f) CLSM images of the corresponding biofilms. (g) Agar plate images and (h) bacterial killing efficiency of CN2, ZT, and ZTCN2 against planktonic bacteria after 2 h of light irradiation. (i, j) Molecular dynamics (MD) snapshots and membrane‐density maps of the planar ZTCN2 and nano‐dagger ZTCN2 model interacting with a bacterial lipid bilayer. (k) Bacterial membrane permeability after treatment under different operating conditions. (l) Interaction force and interaction energy between the nano‐dagger structure and the bacterial membrane obtained from MD simulations. Data are presented as the mean ± SD (n = 3).

SEM was employed to examine bacterial morphology and biofilm architecture following treatment under different operating conditions. On bare Ti under static dark conditions, a dense *P. aeruginosa* biofilm completely covered the surface. The bacterial cells were interconnected by a continuous EPS network, which obscured the underlying substrate and confirmed the poor antibiofilm capability of untreated Ti (Figure S25a). Under electrically driven dynamic actuation, the PHZTCN2 interface substantially disrupted the biofilm architecture through repeated mechanical penetration. Nevertheless, considerable amounts of bacterial debris and organic foulants remained attached to the nano‐dagger array, partially shielding the micro/nanostructures and reducing the accessible sites for ROS generation (Figure 5c). In contrast, under light irradiation alone, ROS effectively inactivated bacteria and oxidatively degraded adhered organic residues. However, because the nano‐dagger array remained mechanically static, localized bacterial aggregates were still observed on the interface (Figure S25b). The most pronounced biofilm removal was achieved under the light‐mechanical coupled mode. Bacterial attachment was almost completely suppressed, and residual proteins and polysaccharides were scarcely detectable. Only a few fragmented bacterial remnants, resulting from membrane penetration, remained on the surface, demonstrating that dynamic mechanical penetration and photo‐induced ROS generation act synergistically to disrupt mature biofilms while continuously removing accumulated foulants. This synergistic mechanism accounts for both the superior antibiofilm efficacy and the excellent long‐term operational stability of the dynamic offensive bio‐interface (Figure 5d).

Fluorescence staining of proteins and polysaccharides was further performed to evaluate the removal of EPS residues from the nano‐dagger interface. As expected, the untreated control exhibited intense EPS‐associated fluorescence, indicating extensive accumulation of organic foulants (Figure S26). Dynamic mechanical penetration alone reduced the fluorescence intensity to some extent; however, substantial protein and polysaccharide residues remained on the PHZTCN2 surface. In contrast, under the light‐mechanical coupled mode, the fluorescence signals of both proteins and polysaccharides were almost completely eliminated, consistent with the SEM observations (Figure 5e,f). These results demonstrate that photo‐induced ROS, together with dynamic mechanical actuation, efficiently remove extracellular matrix components and dead‐cell debris, continuously exposing the active nano‐dagger structures and preventing surface passivation. This self‐cleaning capability provides the basis for the sustained antibiofilm performance and long‐term operational stability of the dynamic offensive bio‐interface.

The antibacterial activities of CN2, ZT, and ZTCN2 against planktonic *P. aeruginosa* were further evaluated to determine whether heterostructure formation could enhance photo‐induced microbial inactivation. Under light irradiation, all three materials exhibited bactericidal activity; however, their inactivation kinetics differed markedly. ZTCN2 showed the fastest antibacterial response, reducing bacterial viability to approximately 7.1% within 30 min and achieving nearly complete inactivation after 60 min. In comparison, ZT required 90–120 min to achieve near‐complete bacterial elimination, with approximately 23.9% of bacteria remaining viable after 60 min. By contrast, CN2 displayed only limited antibacterial activity, with approximately 32.8% of bacteria surviving even after 120 min of illumination. Plate‐counting assays were fully consistent with the bacterial viability results, confirming that construction of the ZTCN2 heterostructure markedly enhances photo‐induced bacterial inactivation (Figure 5g,h and Figure S27) [55]. This enhanced performance is attributed to more efficient photoinduced charge separation and the resulting increase in ROS generation following heterostructure formation. To further elucidate the origin of the enhanced photoactivity, the electronic band structures of CN2 and ZT were investigated using UV–vis spectroscopy and ultraviolet photoelectron spectroscopy (UPS). The optical band gaps, determined from Kubelka‐Munk‐transformed UV–vis spectra, were estimated to be approximately 2.74 eV for CN2 and 1.52 eV for ZT (Figure S28a,b) [56]. UPS measurements yielded secondary electron cutoff energies (*E*_cutoff_) of 15.9 and 16.2 eV for CN2 and ZT, respectively, while both materials exhibited identical Fermi‐edge positions (*E*_Fermi_ = 0 eV). The corresponding work functions (*Φ*) were subsequently calculated according to Equation (4) [55].

(4) $$\Phi =21.22-\left|E_{\mathrm{cutoff}}-E_{\mathrm{fermi}}\right|$$

Based on these values, the work functions of CN2 and ZT were calculated to be 5.32 and 5.02 eV, respectively (Figure S28c,d). Subsequently, the Fermi energy levels of CN2 and ZT were determined from the UPS results according to Equation (5), with the vacuum energy level theoretically differing from the normal hydrogen electrode (NHE) by 4.5 V [57].

(5) $$E_{\mathrm{NHE}}\;\left(\mathrm{vs}.\mathrm{NHE}\right)=\Phi -4.5$$

Accordingly, the Fermi levels of CN2 and ZT were determined to be 0.82 and 0.52 V versus the NHE, respectively. Valence‐band analysis further revealed valence‐band maxima of approximately 3.16 V for CN2 and 0.71 V for ZT (vs. NHE) (Figure S28e,f). If the photogenerated carriers followed a conventional type‐II transfer pathway, electrons and holes would preferentially accumulate on CN2 and ZT, respectively, resulting in insufficient redox potentials for the generation of both ·OH and ·O_2_^−^ radicals. However, ESR measurements detected both ·OH and ·O_2_^−^ radicals under illumination (Figure 3g,h), suggesting that the type‐II charge‐transfer pathway alone cannot fully explain the observed photocatalytic behavior. Taken together with the bandgap and valence‐band analyses, UPS results, and surface‐potential measurements, these findings support the proposal of an S‐scheme charge‐transfer pathway in the ZTCN2 heterostructure.

Before interfacial contact, CN2 and ZT possess different work functions and Fermi levels. Upon contact, electrons are expected to redistribute from the component with the higher Fermi level to that with the lower Fermi level until thermodynamic equilibrium is reached. This process is expected to induce band bending and generate a built‐in interfacial electric field [58]. Under light irradiation, the proposed internal electric field would promote the recombination of low‐energy electrons in CN2 with low‐energy holes in ZT, while preserving the high‐energy electrons in ZT and the strongly oxidative holes in CN2 (Figure S29). This charge‐transfer configuration is consistent with the simultaneous detection of ·OH and ·O_2_^−^ radicals and can explain the enhanced ROS generation observed for the heterostructure [59]. Nevertheless, the built‐in interfacial electric field was not directly spatially resolved or quantitatively measured in this study. Therefore, the S‐scheme pathway is presented here as a proposed mechanistic model supported by the combined spectroscopic, surface‐potential, and radical‐detection results.

To gain molecular‐level insight into the membrane‐disruption mechanism, MD simulations were performed to investigate the interaction between the ZTCN2 nano‐dagger architecture and a model bacterial lipid bilayer (Figure 5i,j). Planar and nano‐dagger ZTCN2 models together with a lipid bilayer were constructed for comparison (Figures S30 and S31). Time‐resolved simulation snapshots revealed that the interfacial interaction was strongly governed by surface geometry. The planar model remained predominantly adsorbed on the membrane surface throughout the simulation, inducing only slight membrane undulations and shallow local indentation without appreciable insertion. In contrast, the nano‐dagger architecture exhibited a characteristic tip‐first penetration pathway, producing pronounced local indentation at the initial stage and progressively embedding into the lipid bilayer, thereby causing substantially greater membrane deformation (Figure 5i). This geometry‐dependent penetration behavior indicates that the sharp nano‐dagger tip generates localized stress concentration, effectively lowering the energetic barrier for membrane insertion and promoting defect formation within the lipid bilayer. Consistently, top‐view lipid‐density maps showed a pronounced reduction in lipid density at the nano‐dagger contact region after 1000 ps, whereas the planar model induced only minor changes (Figure 5j and Figures S32 and S33).

To determine whether dynamic mechanical penetration and photo‐induced ROS synergistically enhance bacterial membrane disruption, membrane‐permeability assays were performed following treatment under different operating conditions, with bare Ti serving as the control. Under static dark conditions, bacteria exposed to PDMS and PH10 exhibited membrane permeability comparable to that of the Ti control, indicating that these surfaces caused negligible membrane damage in the absence of nano‐dagger structures and photo‐induced ROS. Under electrically driven dynamic actuation, membrane permeability increased relative to the corresponding static condition, suggesting that periodic mechanical perturbation imposed additional stress on bacterial cells, although the overall effect remained modest (Figure S34). In contrast, treatment with PHZTCN2 under either static dark or static light conditions resulted in appreciable increases in membrane permeability, demonstrating that both the nano‐dagger architecture and ROS generation independently contribute to membrane damage. Dynamic mechanical penetration produced a substantially greater increase, confirming that active penetration disrupts bacterial membranes and biofilm architecture more effectively than conventional static interfaces. The most pronounced membrane disruption was achieved under the light–mechanical coupled mode, where membrane permeability increased by approximately 5.3 fold compared with the Ti control (Figure 5k). These results demonstrate that dynamic penetration and photo‐induced oxidative stress act synergistically to maximize membrane damage, thereby facilitating efficient disassembly of mature biofilms.

Energetic analysis further supported these observations. The nano‐dagger architecture exhibited a van der Waals interaction energy of 1028.2 kJ mol^−1^, approximately 2.6 times greater than that of the planar model, reflecting its deeper insertion into the lipid bilayer, where van der Waals interactions dominated over electrostatic interactions (Figure 5l and Figure S35). Force analysis likewise demonstrated a marked enhancement in membrane‐disruptive capability. The nano‐dagger generated a penetration force of 17.5 × 10^3^ kJ mol^−1^ nm^−1^, corresponding to approximately 29.1 nN under static conditions, whereas the planar structure produced only ∼5.2 nN. Given that bacterial membrane rupture typically requires forces of 7–25 nN [54], the nano‐dagger geometry is expected to efficiently penetrate bacterial membranes and induce irreversible mechanical damage (Figure 5l and Figure S36). Although the simplified simulation model cannot fully capture the complexity of native biofilm environments, the MD simulations unequivocally reveal the superior membrane‐disruption capability of the nano‐dagger architecture. Combined with the experimental observations, these results demonstrate that the synergistic coupling of electrically actuated dynamic penetration and photo‐responsive ROS‐mediated oxidative stress constitutes the fundamental mechanism driving the active disassembly of mature biofilms.

## 3 Conclusion

In summary, we have developed a porcupine‐spine‐inspired, photo‐responsive dynamic offensive bio‐interface (PHZTCN2) that actively dismantles mature biofilms through the synergistic integration of electrically actuated mechanical penetration and light‐driven oxidative stress. By combining a high‐aspect‐ratio nano‐dagger architecture with reversible electromechanical actuation and enhanced photoactivity, the interface overcomes the intrinsic limitations of conventional passive antibiofilm surfaces. Structural, photoelectrochemical, electromechanical, and antibiofilm characterizations, together with molecular dynamics simulations, demonstrate that PHZTCN2 undergoes reversible nanoscale deformation (∼110 nm) and generates a penetration force of 88.5 µN under an alternating electric field (30 V, 0.5 Hz), corresponding to an approximately 440 fold increase over representative static or weakly dynamic counterparts, resulting in nearly complete eradication of mature *P. aeruginosa* biofilms. The coupled light–mechanical mechanism not only inhibits biofilm formation but also actively dismantles established biofilms while maintaining stable antibiofilm performance for at least 10 days without chemical biocides, extending representative previously reported operational durations by approximately 43%. More importantly, this work establishes an active antibiofilm paradigm that shifts interfacial protection from passive resistance to dynamic biofilm disassembly. Beyond antibiofilm applications, this offensive bio‐interface provides a general design framework for engineering durable self‐cleaning and antifouling surfaces capable of long‐term operation in complex environments. It should be noted that only *P. aeruginosa* was evaluated in this study. Therefore, the present results are limited to the model *P. aeruginosa* biofilm system. Further validation using multispecies biofilms under more realistic marine conditions will be necessary to assess the practical applicability of this interface for marine antifouling applications.

## 4 Experimental Section

The fabrication process of the photo‐responsive dynamic nano‐dagger interface is illustrated in Figure S37. ZT was prepared as described in the Supporting Information. HfO_2_ nanoparticles prepared by hydrothermal calcination were dispersed in PDMS at a loading of 10% (w/v) and uniformly coated onto the Ti substrate to form the dielectric elastomer layer. After curing, the substrate was immersed in a precursor solution containing CoCl_2_·6H_2_O, NiCl_2_·6H_2_O, Zn‐TCPP, and urea for hydrothermal growth of the photo‐responsive ZTCN2 nano‐dagger array. Subsequently, the resulting surface was rendered conductive by ion sputtering to form the top conductive electrode. The final PHZTCN2 device consisted of a Ti substrate, an HfO_2_‐modified PDMS dielectric layer, a ZTCN2 nano‐dagger layer, and a sputter‐deposited top conductive electrode. Detailed information regarding the materials, synthesis procedures, fabrication process, structural characterization, and antibiofilm evaluation is provided in the Supporting Information.

## Author Contributions

**Feng Qian**: methodology, Writing – original draft, software. **Muqiu Xia**: methodology, formal analysis. **Ziyu Wang**: methodology, formal analysis. **Xinyuan Jia**: formal analysis. **Jinke Yin**: formal analysis. **Mengyu Fu**: formal analysis. **Xue Zhang**: formal analysis. **Xiao‐Lei Shi**: writing – review and editing. **Shiqiang Chen**: conceptualization, data curation, investigation, validation, supervision, resources, project administration, funding acquisition. **Ning Wang**: funding acquisition, project administration, resources, conceptualization. **Zhi‐Gang Chen**: conceptualization, writing – review and editing, project administration, data curation, formal analysis, investigation.

## Conflicts of Interest

The authors declare no conflict of interest.

## Supporting information




**Supporting File**: advs78251‐sup‐0001‐SuppMat.pdf.

## Data Availability

The data that support the findings of this study are available from the corresponding author upon reasonable request.
