# Supplementary material for: Porcupine‐Quill‐Inspired Shape‐Morphing Biointerfaces Disassemble Biofilms
Source: Adv Sci (Weinh). 2026 Oct 12:e78251. Online ahead of print. doi: 10.1002/advs.78251 (PMC13658894; doi:10.1002/advs.78251)
Supplement: Supplementary file 1 [file ADVS-9999-e78251-s001.pdf]

Supporting Information

Porcupine-Quill-Inspired Shape-Morphing Biointerfaces Disassemble Biofilms

Feng Qian [a, d], Muqiu Xia [a, d], Ziyu Wang [a, d], Xinyuan Jia [a, d], Jinke Yin [a, d], Mengyu Fu [a, d], Xue Zhang [a, d], Xiao-Lei Shi [c], Shiqiang Chen [b], Ning Wang [b*], Zhi-Gang Chen [c*]*

[a] Institute of Marine Science and Technology, Shandong University, Qingdao, Shandong, 266237, China;

[b] College of Chemical Engineering, Qingdao University of Science and Technology, 53 Zhengzhou Road, Qingdao 266042, Shandong, China;

[c] School of Chemistry and Physics and Centre for Materials Science, ARC Research Hub in Zero-emission Power Generation for Carbon Neutrality, Queensland University of Technology, Brisbane, Queensland, Australia;

[d] Shandong Key Laboratory of Intelligent Marine Engineering Geology, Environment and Equipment, Institute of Marine Science and Technology, Shandong University, Qingdao 266237, Peoples R China.

1 Supplementary Experimental Section

1.1 Materials and Reagents.

All chemical reagents used in this study were of analytical grade and used as received without further purification. TA1 titanium sheets (titanium content ≥99.2%) were purchased from Taizhou Tide Co., Ltd. (Taizhou, China) and cut into pieces of 10 mm × 10 mm × 0.5 mm. Cobalt (II) chloride hexahydrate ($CoCl_2·6H_2O$), nickel (II) chloride hexahydrate ($NiCl_2·6H_2O$), urea (H_2NCONH_2), cetyltrimethylammonium bromide ($C_{19}H_{42}BrN$), and meso-tetrakis(4-carboxyphenyl)porphyrin ($C_{48}H_{30}N_4O_8$) were purchased from Aladdin Reagent Co., Ltd. (Shanghai, China). Zinc acetate dihydrate ($C_4H_6O_4Zn·2H_2O$), sodium hydroxide (NaOH), ethanol (C_2H_6O), and hydrochloric acid (HCl) were obtained from Sinopharm Chemical Reagent Co., Ltd. (Shanghai, China). Hafnium (IV) chloride ($HfCl_4$) was supplied by Shanghai Macklin Biochemical Co., Ltd. (Shanghai, China). Polydimethylsiloxane (PDMS) was purchased from Dow Corning Corporation.

1.2 Synthesis of HfO_2 nanoparticle, PDMS coatings and PDMS coatings modified by HfO_2.

HfO_2 nanoparticles were prepared via a hydrothermal-calcination method. Specifically, 50 mM $HfCl_4$ was added to 88 mL of 3 M NaOH solution and stirred for 20 min until the solution became milky. The mixture was then transferred into a 100 mL Teflon-lined autoclave and reacted at 180°C for 12 h in a forced-air drying oven. After the reaction, the precipitate was collected by centrifugation and washed repeatedly with deionized water. The product was dried in a forced-air oven at 60°C until completely dry. Finally, the dried powder was calcined in a muffle furnace at 700°C for 2 h, and the resulting powder was collected to obtain HfO_2 nanoparticles.

PDMS was prepared by a two-component curing process. The prepolymer and curing agent were mixed at a mass ratio of 20:1 and thoroughly blended using a mixer at 1500 rpm. The mixture was

then uniformly coated onto the Ti surface using a film applicator, followed by curing in a forced-air oven at 80°C for 6 h. After curing, Copper wires were used for electrical connection, and the wire-connection regions were fixed using conductive adhesive. The back side, peripheral edges, and electrical connection regions were sealed with 703 silicone sealants to isolate these components from the aqueous medium. Only the defined 1 cm^2 functional surface was exposed during the antibiofilm tests. Finally, a conductive top layer was deposited by ion sputtering after hydrothermal growth of the ZTCN2 nano-dagger array. The conductive paste was used only for electrical connection of the copper wire and was sealed with 703 silicone sealant.

The preparation of HfO_2-modified PDMS surfaces followed the same procedure, except that HfO_2 nanoparticles were added during the mixing of the two PDMS components at different mass-to-volume ratios. The resulting surfaces were denoted as PH*x* (*x* = 1, 5, 10, and 15), corresponding to the different HfO_2 loadings.

1.3 Preparation of $NiCo(OH)_2CO_3$ nano dagger arrays and modification of Zn-TCPP

$NiCo(OH)_2CO_3$ nano-dagger arrays were grown on PHx surfaces via a hydrothermal method. In brief, 166 mM $CoCl_2·6H_2O$, 83 mM $NiCl_2·6H_2O$, and 200 mM urea were added to 15 mL deionized water and stirred for 30 min until completely dissolved, yielding a purplish-red solution. The solution was then transferred into a 50 mL Teflon-lined autoclave. The as-prepared PH*x* substrate was placed inside the liner in a tilted position, and the hydrothermal reaction was carried out at 120°C for 6 h in a forced-air oven. After the reaction, the substrate was removed, rinsed alternately with deionized water and ethanol, and air-dried to obtain the nano-dagger array modified surface. Under these conditions, the Co:Ni atomic ratio was 2:1. Subsequently, by increasing the $CoCl_2·6H_2O$ concentration to 333 mM and 666 mM (while keeping $NiCl_2·6H_2O$ at 83 mM), nano-dagger arrays with different Co:Ni ratios were obtained and denoted as PHCN*y* (*y* = 2, 4, and 8). For nano-dagger arrays prepared separately on

FTO conductive glass, the samples were denoted as CN*y* (*y* = 2, 4, and 8).

Zn-TCPP was prepared via a self-assembly method, adapted from the work of Zhu *et al.* with minor modifications [1]. Specifically, 15 mM $C_{48}H_{30}N_4O_8$ was added to 60 mL of 0.1 M NaOH solution, followed by the addition of 19 mM $C_4H_6O_4Zn·2H_2O$. The mixture was heated under reflux at 120°C for 2 h to allow sufficient reaction and self-assembly. After completion, the reaction mixture was cooled to room temperature, and the resulting cooled solution was denoted as solution C. Solution D was then prepared by dissolving 2 mM $C_{19}H_{42}BrN$ in 500 mL deionized water, followed by dropwise addition of concentrated HCl to reach a final HCl concentration of 12 mM. Subsequently, solution C was rapidly poured into solution D and stirred at room temperature for 24 h under light-protected conditions. The product was collected by repeated washing with deionized water and centrifugation at 8000 rpm, and then dried in a vacuum oven at 70°C to obtain Zn-TCPP nanomaterials.

Zn-TCPP/$NiCo(OH)_2CO_3$ was prepared by adding Zn-TCPP nanomaterials into the precursor solution used for the growth of $NiCo(OH)_2CO_3$ nano-dagger arrays, while keeping all other experimental steps and conditions unchanged.

1.4 Characterization

The surface morphologies of different materials were examined using a field-emission scanning electron microscope (SEM, FEI Quanta 250 FEG). For observing the micro-morphology of microorganisms, sample pretreatment was required: the samples were first fixed in 2.5% glutaraldehyde solution for 2 h and then dehydrated using a graded ethanol series. High-resolution morphology and lattice information of the materials were further confirmed by transmission electron microscopy (TEM, JEM-2100PLUS).

The crystal structures and elemental valence states were analyzed by X-ray diffraction (XRD,

D/MAX/2500PC) and X-ray photoelectron spectroscopy (XPS, AXIS SUPRA), respectively. Molecular structures were characterized using a Fourier transform infrared micro spectrometer (FT-IR, Nicolet iN10) and a Raman spectrometer (Raman, InVia Reflex). The dielectric properties were determined by dielectric impedance spectroscopy (DIS, Alpha-A). The surface displacement at different voltages is measured using a laser displacement Instrument coupled with a high-voltage module (LDI, LSV120NG), using a sine wave. The mechanical properties of the films were evaluated using a universal mechanical testing machine (UMTM, JHY-5000) at a testing speed of 50 mm/min. Atomic force microscopy (AFM, Bruker Dimension ICON) was used to analyze the morphology and height of the materials, and the surface potentials under dark and illuminated conditions were measured in KPFM mode. Carrier separation efficiency and carrier lifetime were characterized by photoluminescence spectroscopy (PL, F-7000). Optical absorption properties were measured using a UV-vis-NIR spectrophotometer (UV-vis, Cary 5000). Electron spin resonance spectroscopy (ESR, Bruker EMXplus) was employed to detect radical generation under light irradiation.

1.5 Anti-biofilm tests

Natural seawater used in this study was collected from Qingdao, China. *P. aeruginosa* was cultured in a modified LB medium containing 10 g peptone, 5 g yeast extract, and 1 L natural seawater. The *P. aeruginosa* strain was isolated from the rust layer formed on Q235 carbon steel after immersion in natural seawater for 6 months. The prepared samples were immersed in a culture medium containing *P. aeruginosa* in the logarithmic growth phase (10^9 CFU mL^{-1}). Four groups were set up, including a static group, an electric-field group, a light-irradiation group, and a light-electric field coupled group, to evaluate the antibiofilm performance of the samples. During the experiments, both the electric field and light irradiation were applied intermittently, i.e., applied continuously for 1 h every 3 h. For the long-term experiments, *P. aeruginosa* biofilms were first pre-cultured on the sample surfaces for 1, 5, or 10 days. The culture medium was renewed every 3 days to replenish nutrients and maintain bacterial

growth activity. After the designated biofilm-growth period, the samples were subjected to intermittent light/electrical actuation for 1 h every 3 h. Three treatment cycles were performed before the samples were characterized. Accordingly, the 1-day biofilm represented an early-stage attached biofilm, whereas the 5- and 10-day biofilms represented more established biofilms. After the experiments, bacterial viability on the sample surfaces was observed using confocal laser scanning microscopy (CLSM, LSM 880). Because direct in situ CLSM imaging of the electrode surface was affected by substrate roughness, opacity, reflection, and heterogeneous topography, a standardized detachment-based imaging procedure was used for quantitative analysis. After treatment, each sample was immersed in PBS and vortexed for 5 min to recover surface-associated bacteria. The resulting suspension was transferred onto a glass slide and stained with a fluorescent dye (FilmTracerTM LIVE/DEAD® Biofilm Kit, Thermo Fisher Scientific) for 15 min in the dark. CLSM images were then acquired under identical settings for all groups. The resulting images represent bacteria recovered from the treated surfaces and were used to quantify the relative viable/dead-cell fluorescence signals. Fluorescence intensities were quantified using ImageJ software, and the antibiofilm efficiency was calculated according to the following Equation (S1):

$$\text{Anti} - \text{biofilm rate } (\%) = \frac{(L_{\text{ctrl}} - L_{\text{anti}})}{L_{\text{ctrl}}} \times 100 \qquad (S1)$$

where L_{ctrl} represents the fluorescence intensity of live bacteria on the surface of the control group, and L_{anti} represents the fluorescence intensity of live bacteria on the sample surfaces under different test conditions.

Killing efficiency was calculated according to Equation (S2):

$$\text{Bacterial mortality rate } (\%) = \frac{(L_{\text{total}} - L_{\text{live}})}{L_{\text{total}}} \times 100 \qquad (S2)$$

where L_{total} represents the total bacterial fluorescence intensity on the sample surfaces under different test conditions, and L_{live} represents the fluorescence intensity of live bacteria on the sample surfaces under different test conditions.

For the membrane permeability assay, a bacterial suspension (100 μL, 10^9 CFU mL^{-1}) was inoculated onto the samples and treated under different conditions for 24 h. Then, 900 μL PBS was added and mixed thoroughly. A 1 mM ANS solution was added to the resulting suspension and incubated at 37°C for 20 min. Fluorescence intensity was measured using a microplate reader at λ_{ex} = 380 nm and λ_{em} = 460 nm.

For the experiment on the removal of free bacteria, 10 mL of *P. aeruginosa* cultured to the logarithmic phase (concentration of approximately 10^9 CFU mL^{-1}) was added to 90 mL PBS, and 50 μg of the sample was added to the above bacterial suspension. The photocatalytic free bacteria removal experiment was carried out under magnetic stirring, with circulating cooling water used throughout the experiment to maintain room temperature. Simulated sunlight was obtained using a xenon lamp equipped with an AM 1.5 filter, and the light intensity was calibrated to 100 mW cm^{-2} using a light power meter.

1.6 Electrochemical measurements

All electrochemical measurements were performed using a P2000A electrochemical workstation, with the sample as the working electrode and graphite and Ag/AgCl electrodes as the counter and reference electrodes, respectively. The electrochemically active surface area (ECSA) was determined by cyclic voltammetry (CV) at scan rates of 10, 20, 30, 40, and 50 mV s^{-1}. The electric field was applied in potentiostatic mode using the P2000A workstation, and periodic application of an intermittent electric field was achieved using a loop program.

1.7 Molecular dynamics (MD) simulation

The models were constructed using a combination of Packmol and Python [2]. The systems consisted of nano-dagger, planar, and lipid-bilayer models in an aqueous environment, all maintained under charge-neutral conditions. All-atom molecular dynamics simulations were performed in GROMACS using the CHARMM36m force field [3-4]. The simulated systems were first subjected to energy minimization to obtain a physically reasonable structure. During the dynamic stage, simulations were carried out under periodic boundary conditions (PBC). The systems were gradually heated to 298.15 K, and the pressure was maintained at the target value using a Parrinello-Rahman barostat [5]. Nonbonded interactions were treated with the Verlet cutoff scheme. Long-range electrostatics were calculated using the particle mesh Ewald (PME) method with a grid spacing of 0.16 nm and a Coulomb cutoff of 1.2 nm [6]. Van der Waals interactions were modeled with a simple cutoff of 1.2 nm, and dispersion corrections for energy and pressure were applied. Temperature was controlled using the velocity-rescaling thermostat. The simulations employed the leapfrog integration algorithm with a time step of 1 fs (0.001 ps) and were run for a total of 1 ns. The neighbor list was updated every 10 steps, and hydrogen bonds were constrained using the LINCS algorithm [7]. During the simulations, OVITO and VMD were used for visualization. All simulation data were processed and analyzed using Python-based tools and scripts [8-9].

2 Supplementary Figures

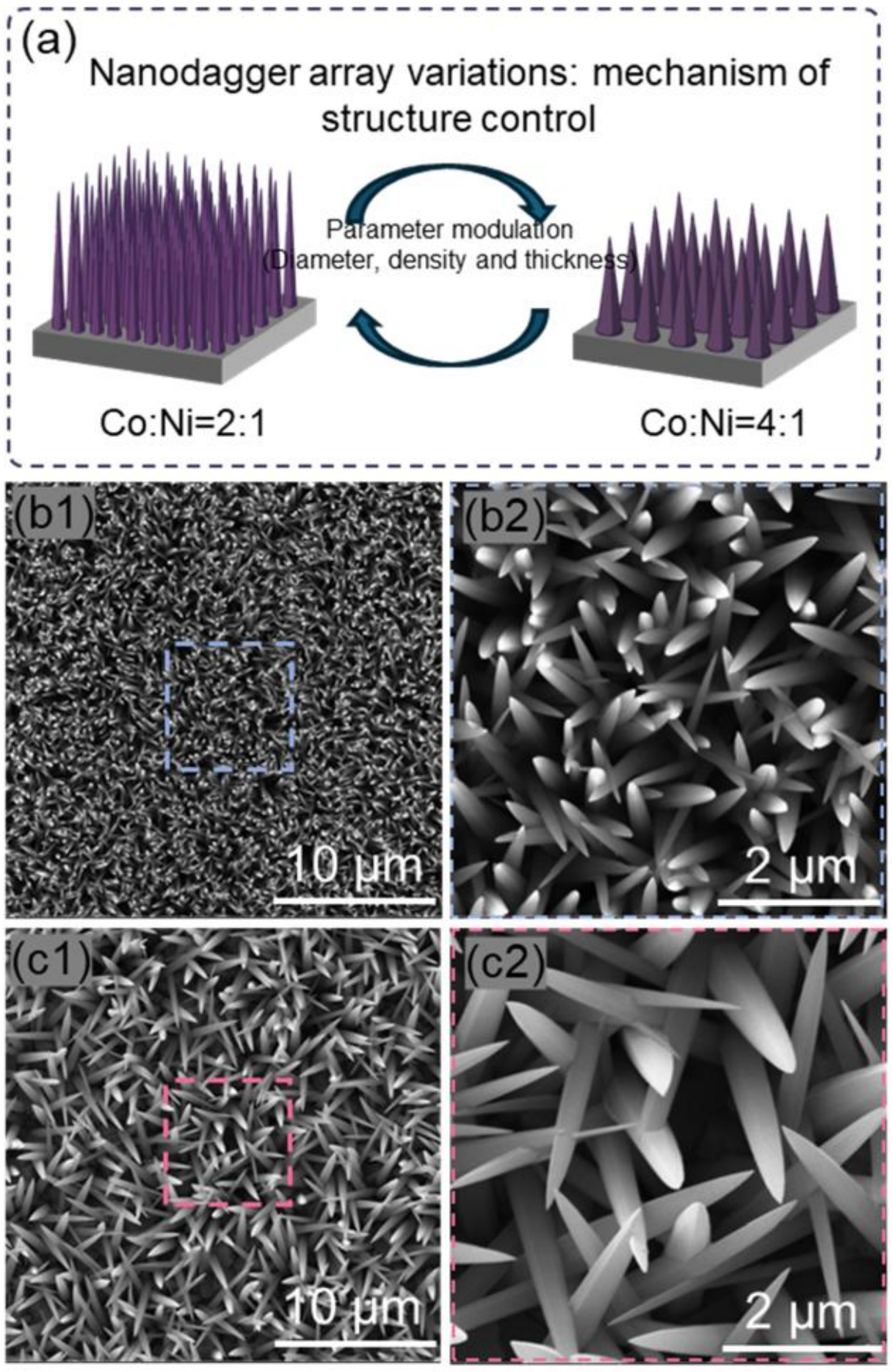

Figure S1. (a) Schematic diagram of the regulated nano-dagger structure; **(b-c)** SEM results of the CN2 and CN4.

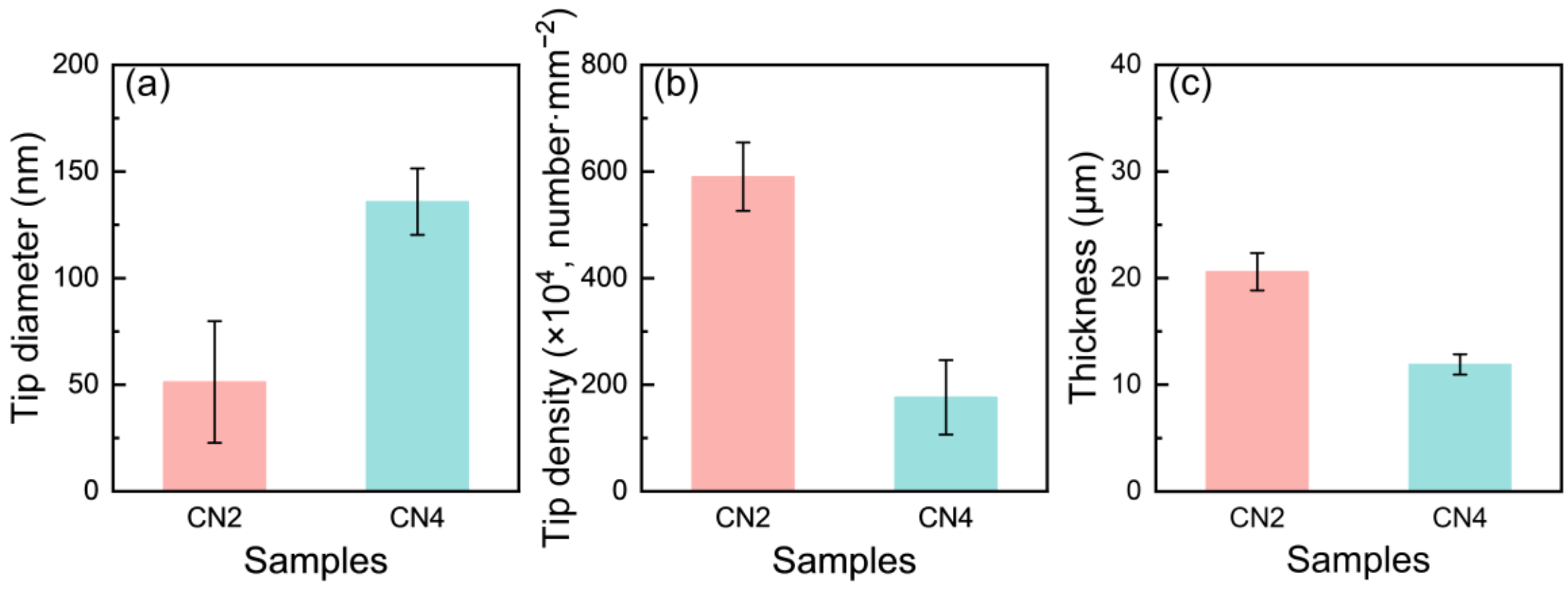

Figure S2. (a) Counting results for the tip diameter, **(b)** tip density, and **(c)** thickness of CN2 and CN4.

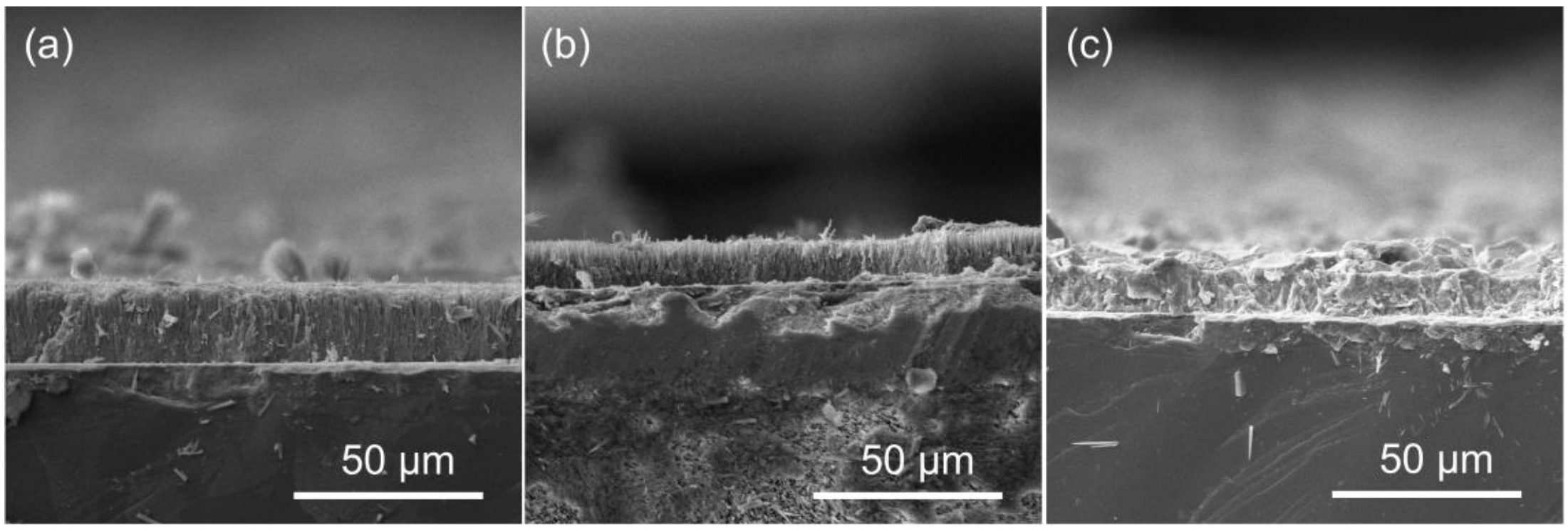

Figure S3. The cross-sectional SEM images of **(a)** CN2, **(b)** CN4 and **(c)** CN8.

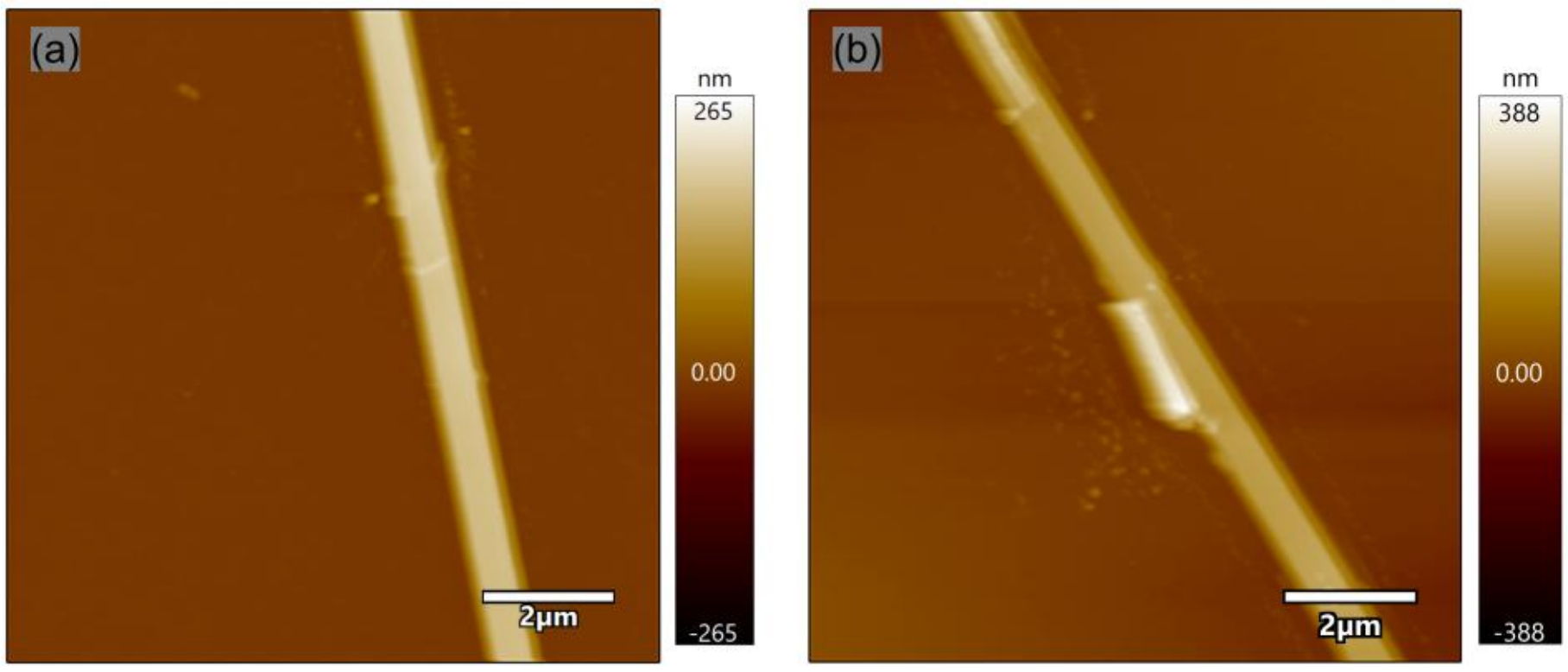

Figure S4. The AFM results of **(a)** CN2 and **(b)** ZTCN2.

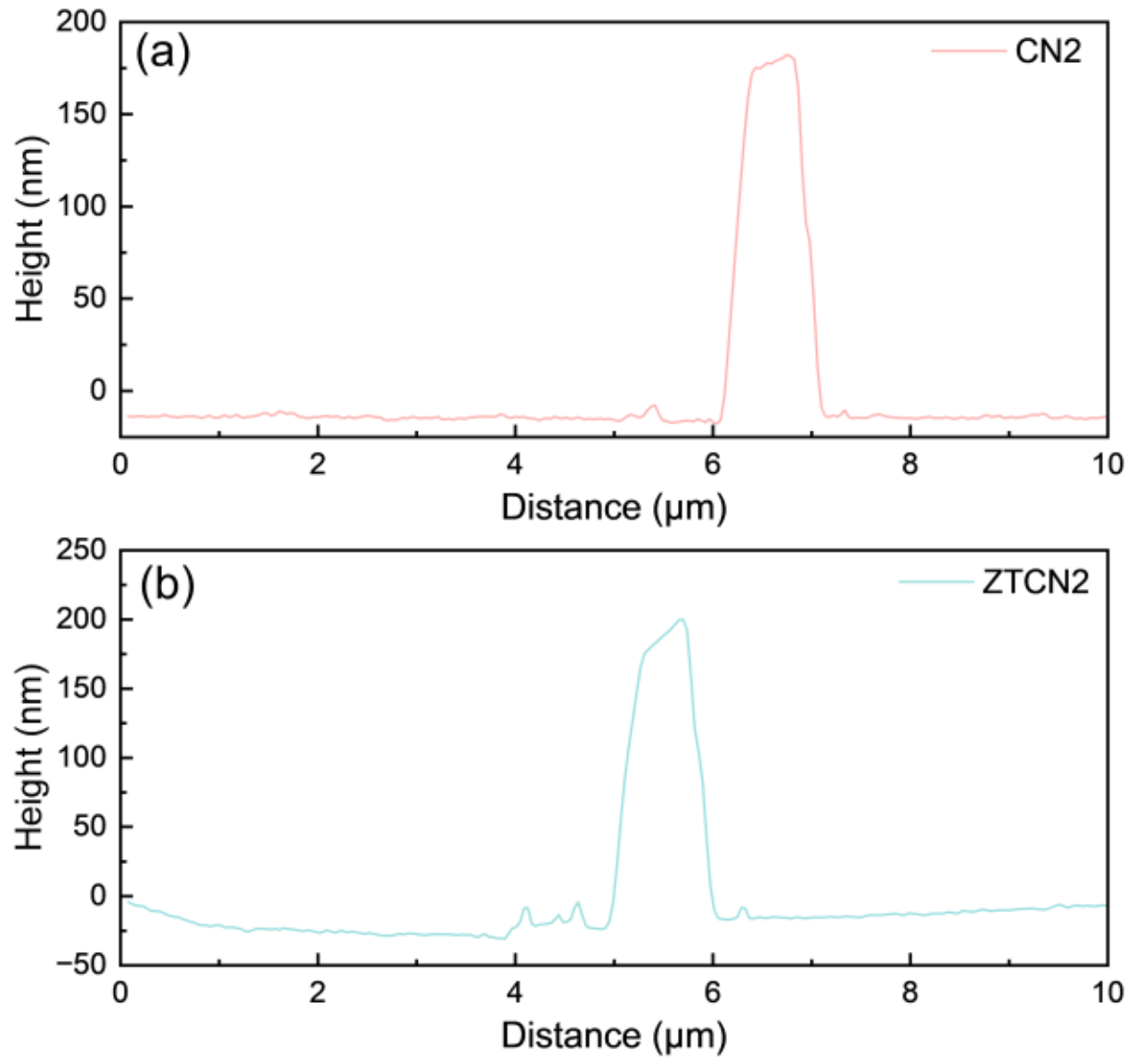

Figure S5. The height results of **(a)** CN2 and **(b)** ZTCN2.

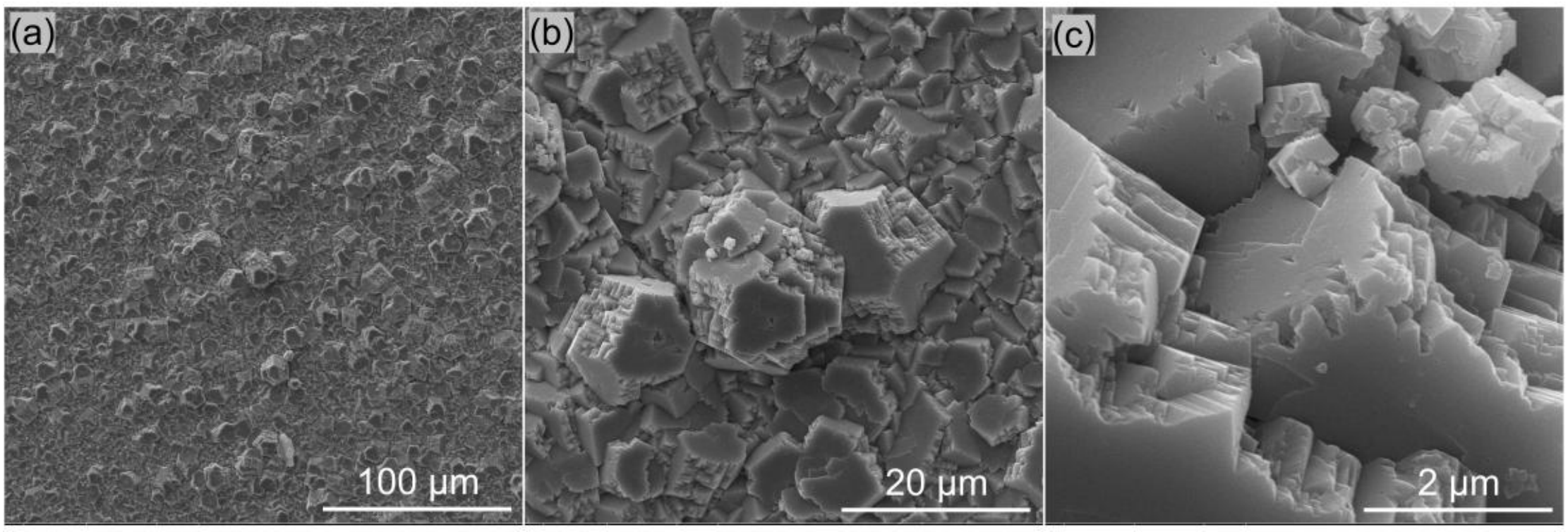

Figure S6. (a-c) SEM results of CN8 at different resolutions.

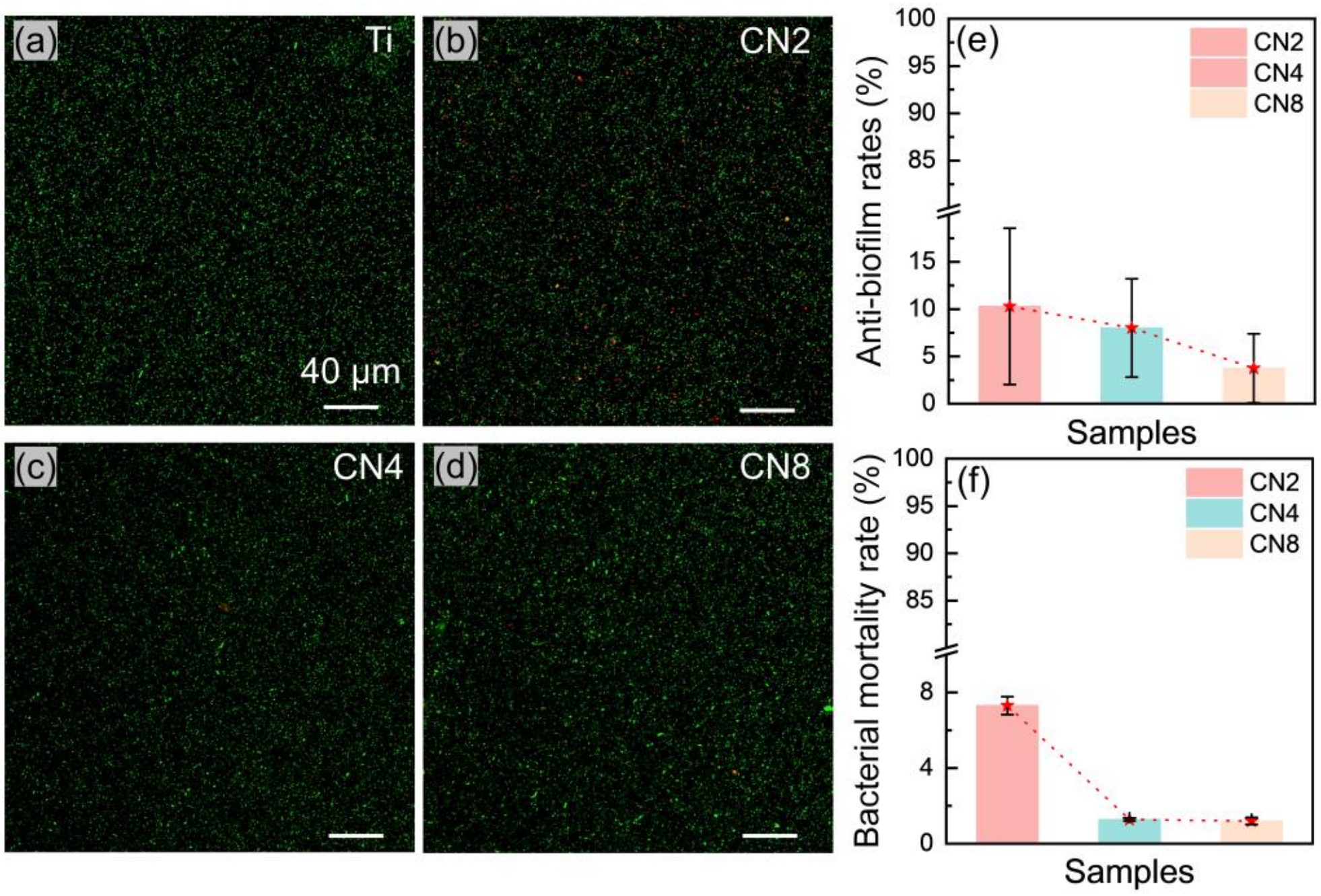

Figure S7. (a-d) CLSM results of different surfaces after being immersed in *P. aeruginosa* seawater for 1 d under static conditions. **(e)** Anti-biofilm rate and **(f)** bacterial mortality rate of different surfaces.

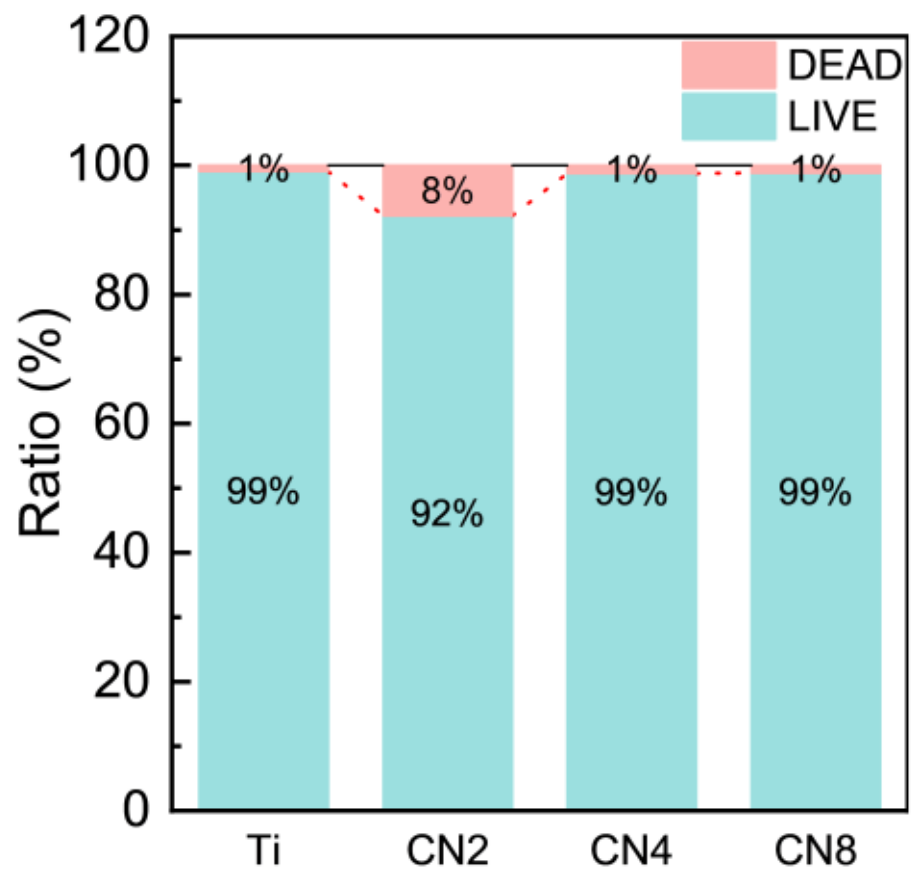

Figure S8. The proportion of live and dead bacteria on different surfaces after being immersed in *P. aeruginosa* seawater for 1 d under static conditions.

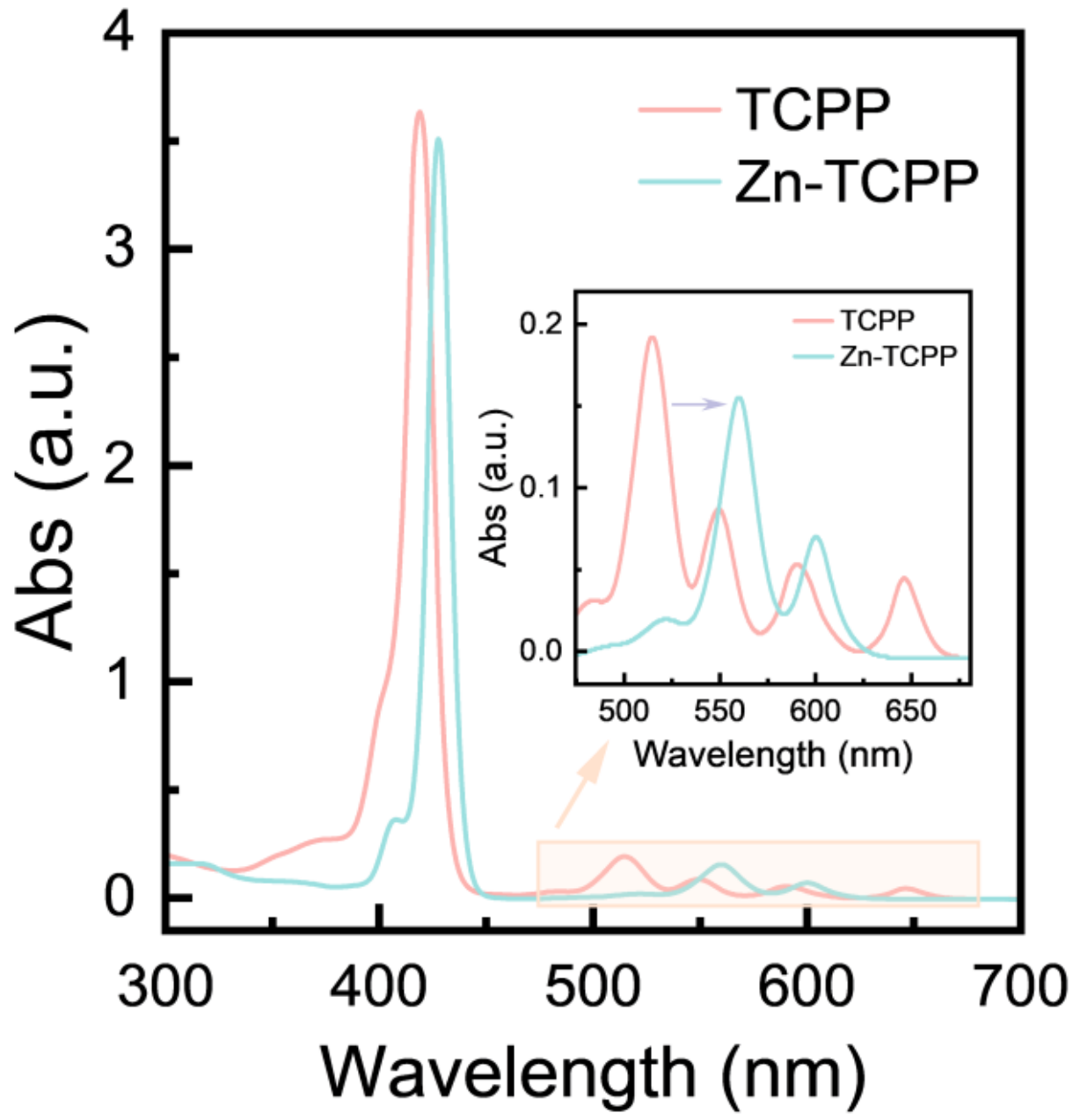

Figure S9. The UV−vis absorption spectra of free-based TCPP and synthesized ZT.

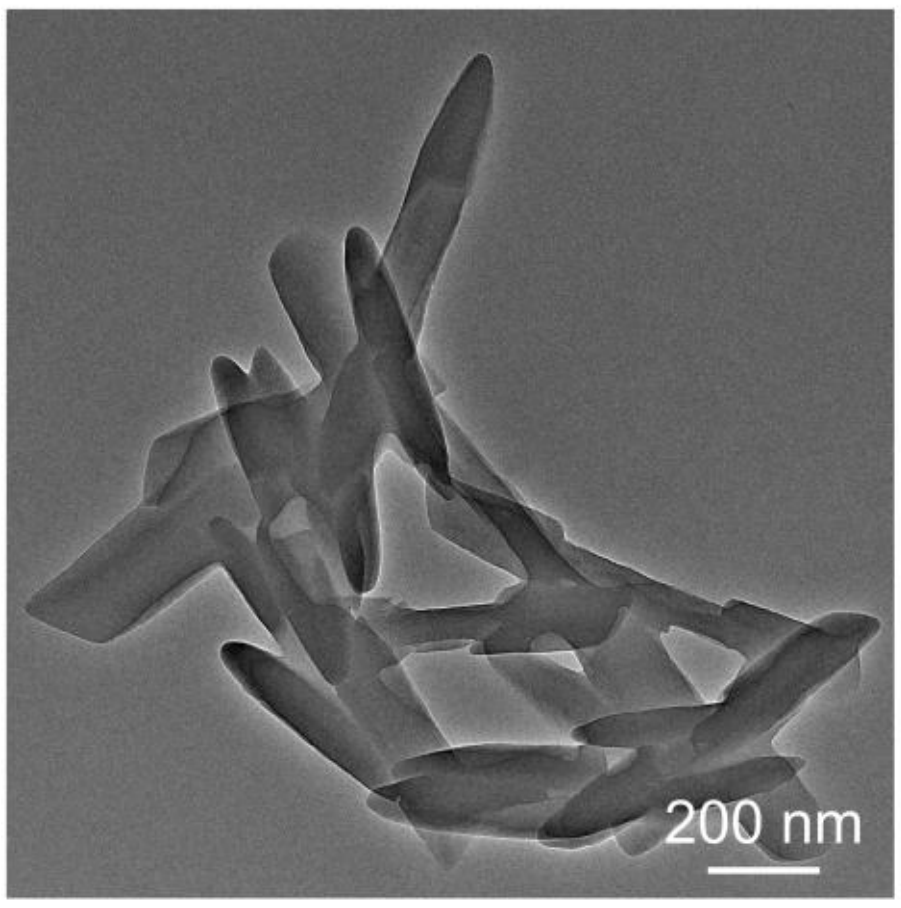

Figure S10. The TEM results of ZT.

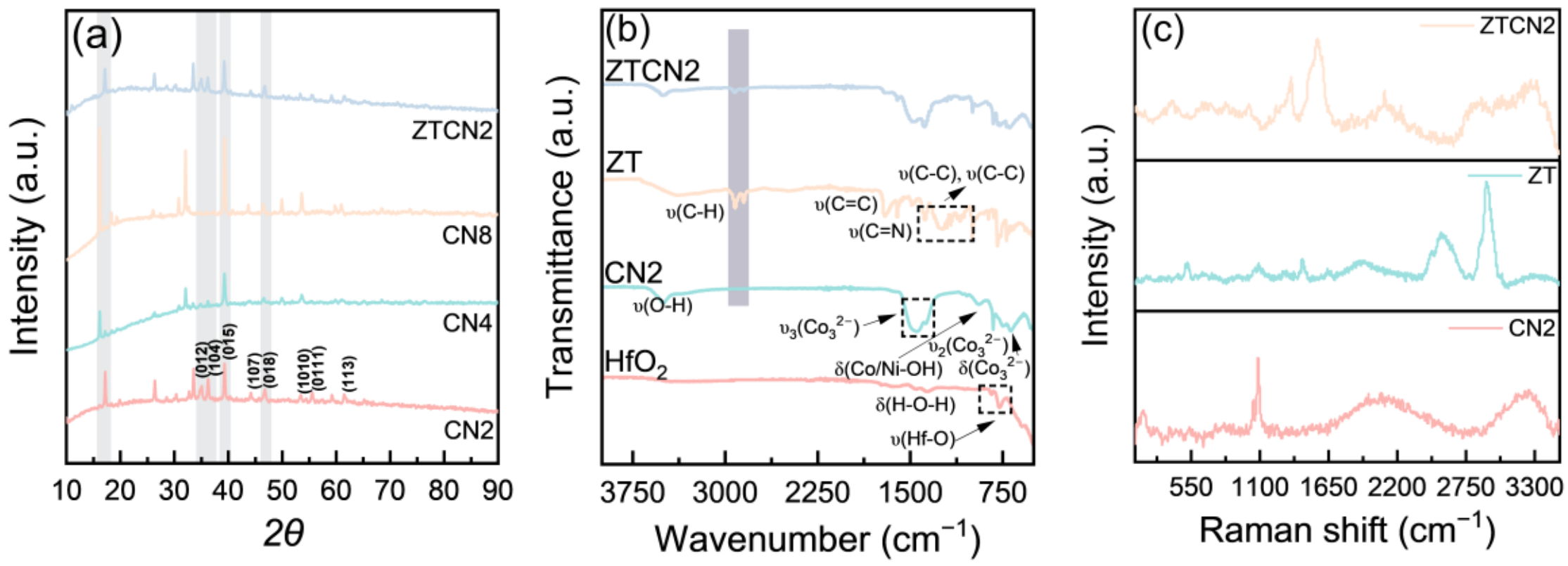

Figure S11. (a) XRD results of CN2, CN4, CN8 and ZTCN2. **(b)** FTIR results of HfO_2, CN2, ZT and ZTCN2. **(c)** Raman results of CN2, ZT and ZTCN2.

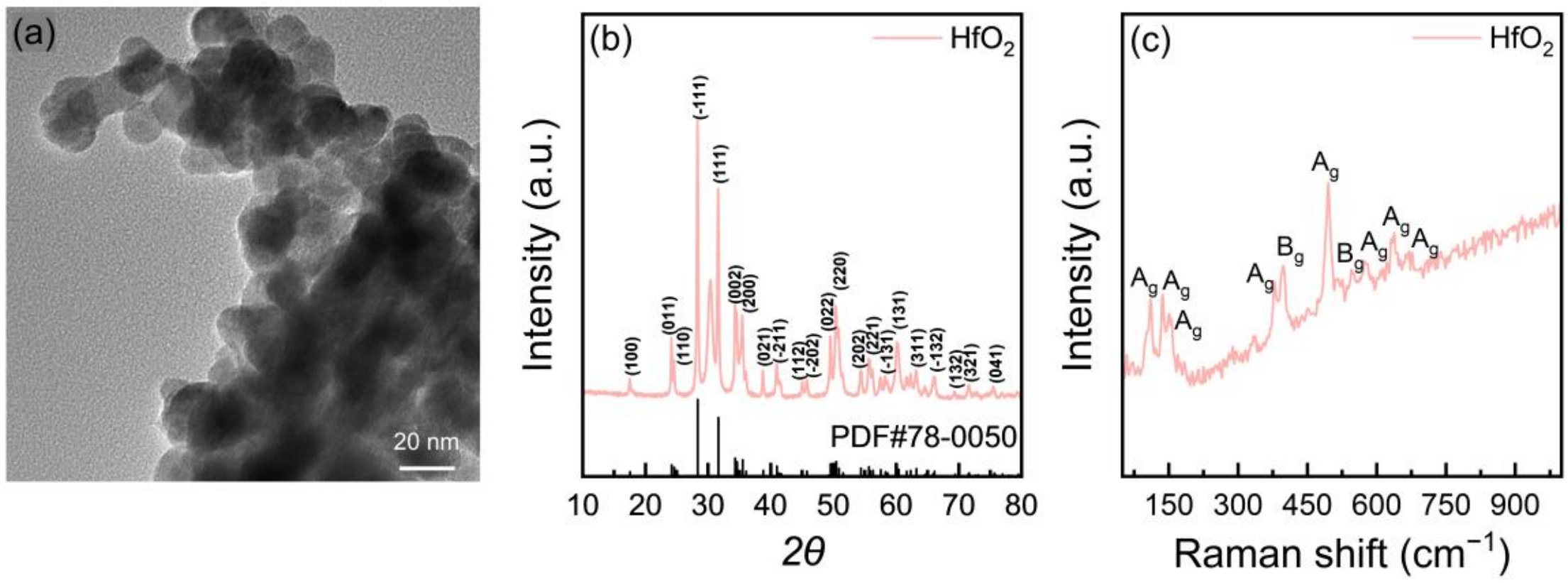

Figure S12. (a) TEM, **(b)** XRD, and **(c)** Raman spectroscopy results of HfO_2.

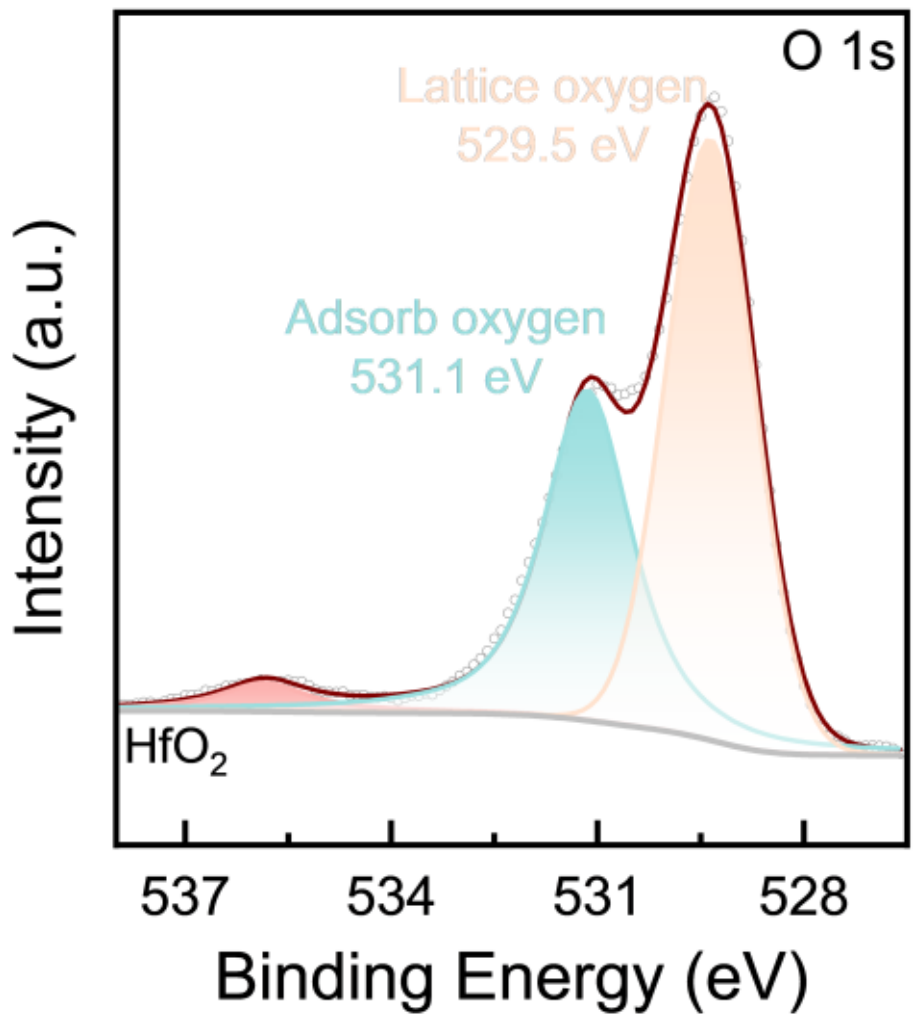

Figure S13. The XPS fine spectra of O results of HfO_2.

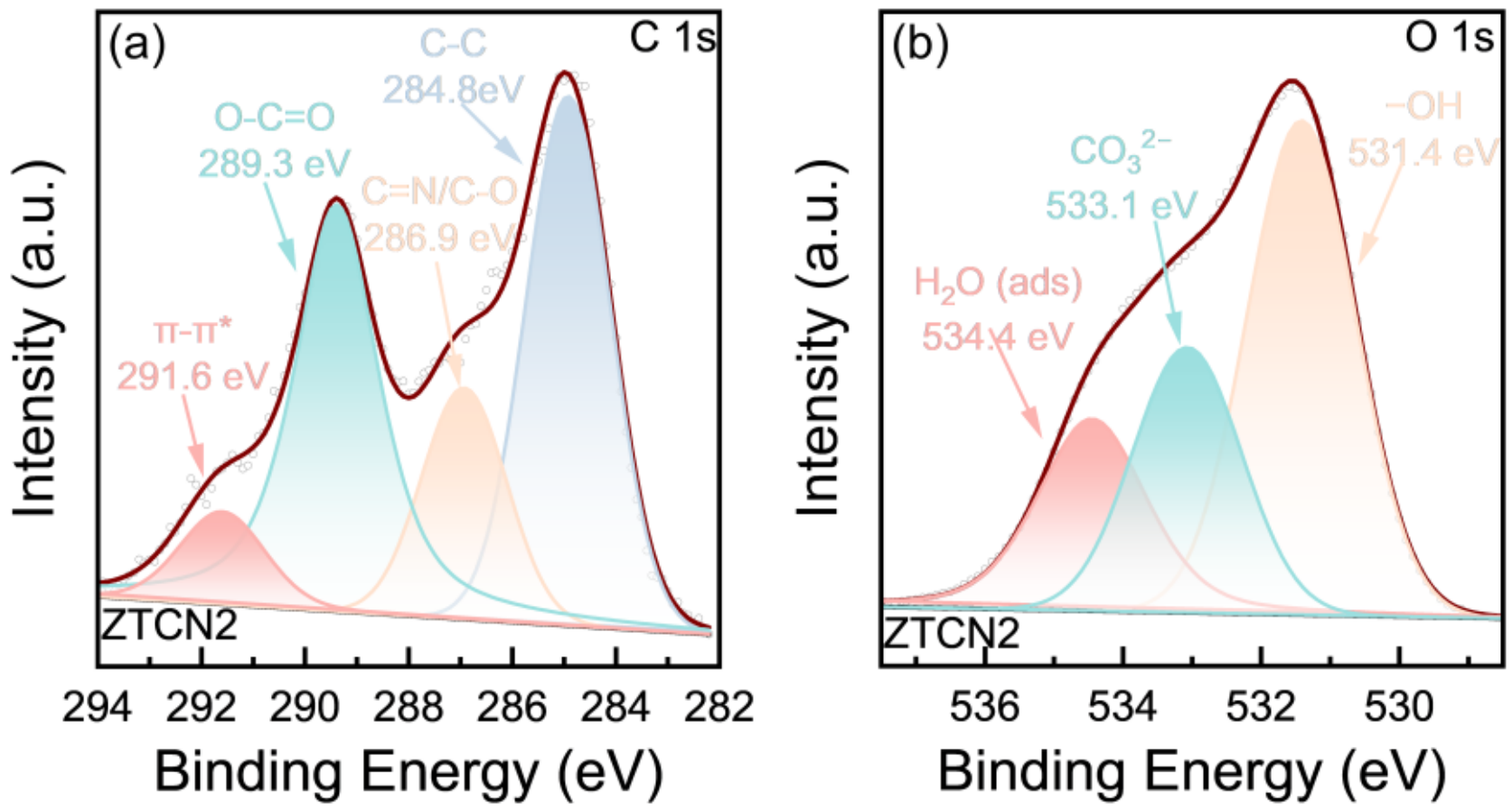

Figure S14. The XPS fine spectra of ZTCN2 for **(a)** C and **(b)** O.

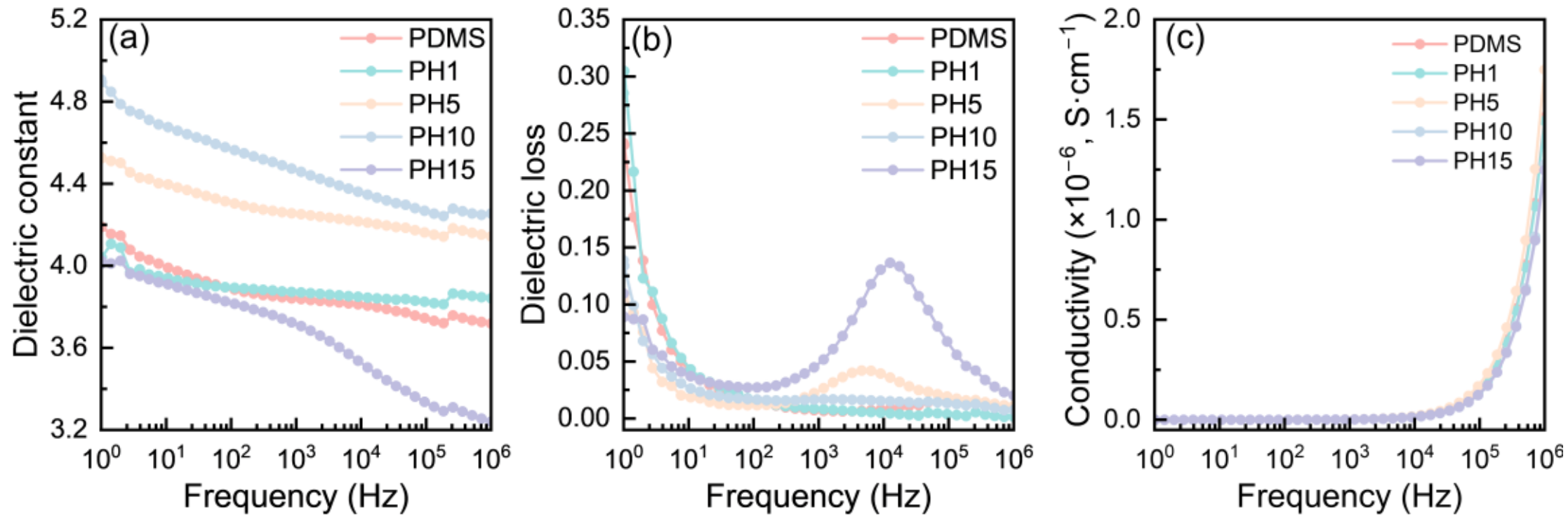

Figure S15. (a) Dielectric constant, **(b)** dielectric loss, and **(c)** conductivity results of PDMS and HfO_2-modified PDMS.

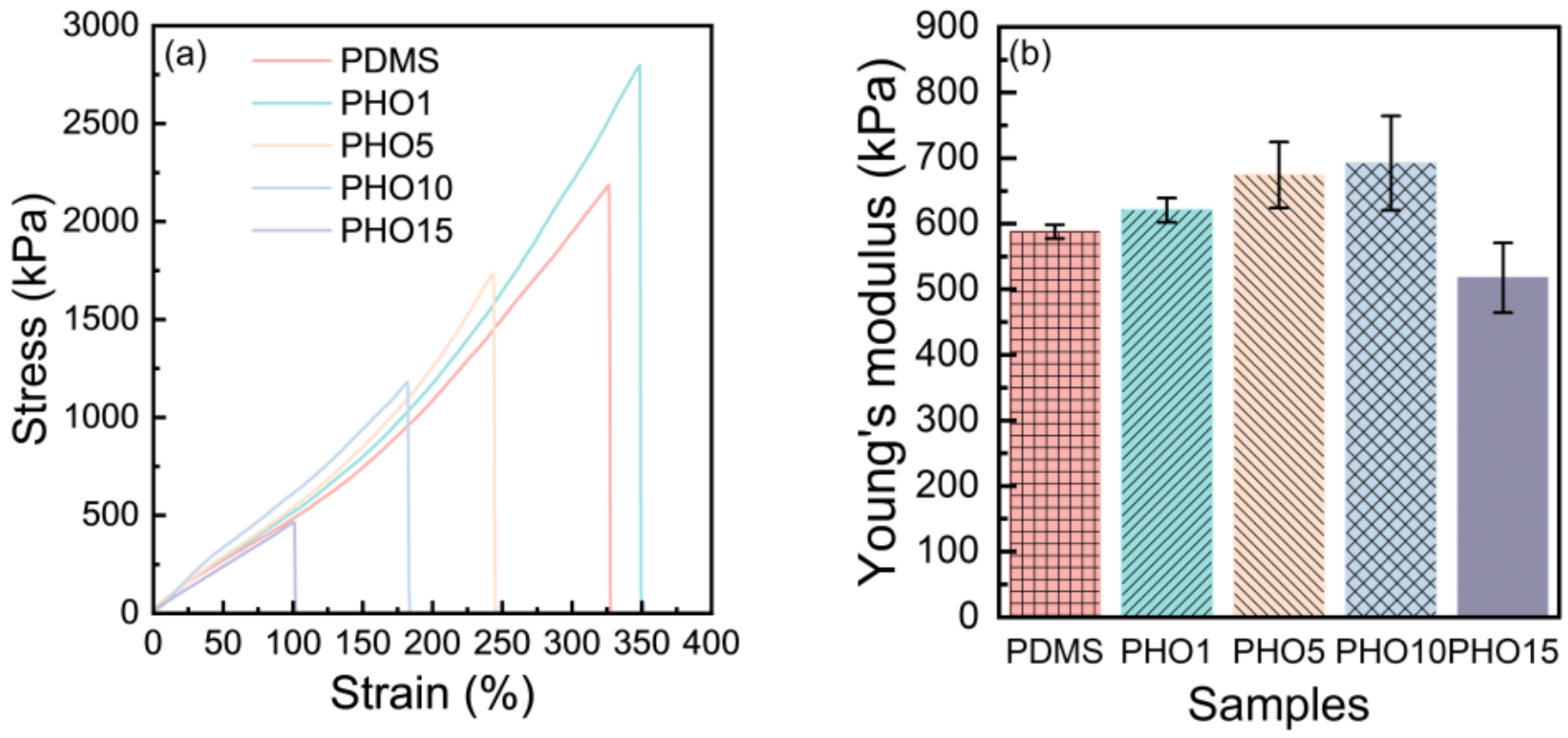

Figure S16. (a) Stress-strain curves and **(b)** Young's modulus results of PDMS and HfO_2-modified PDMS.

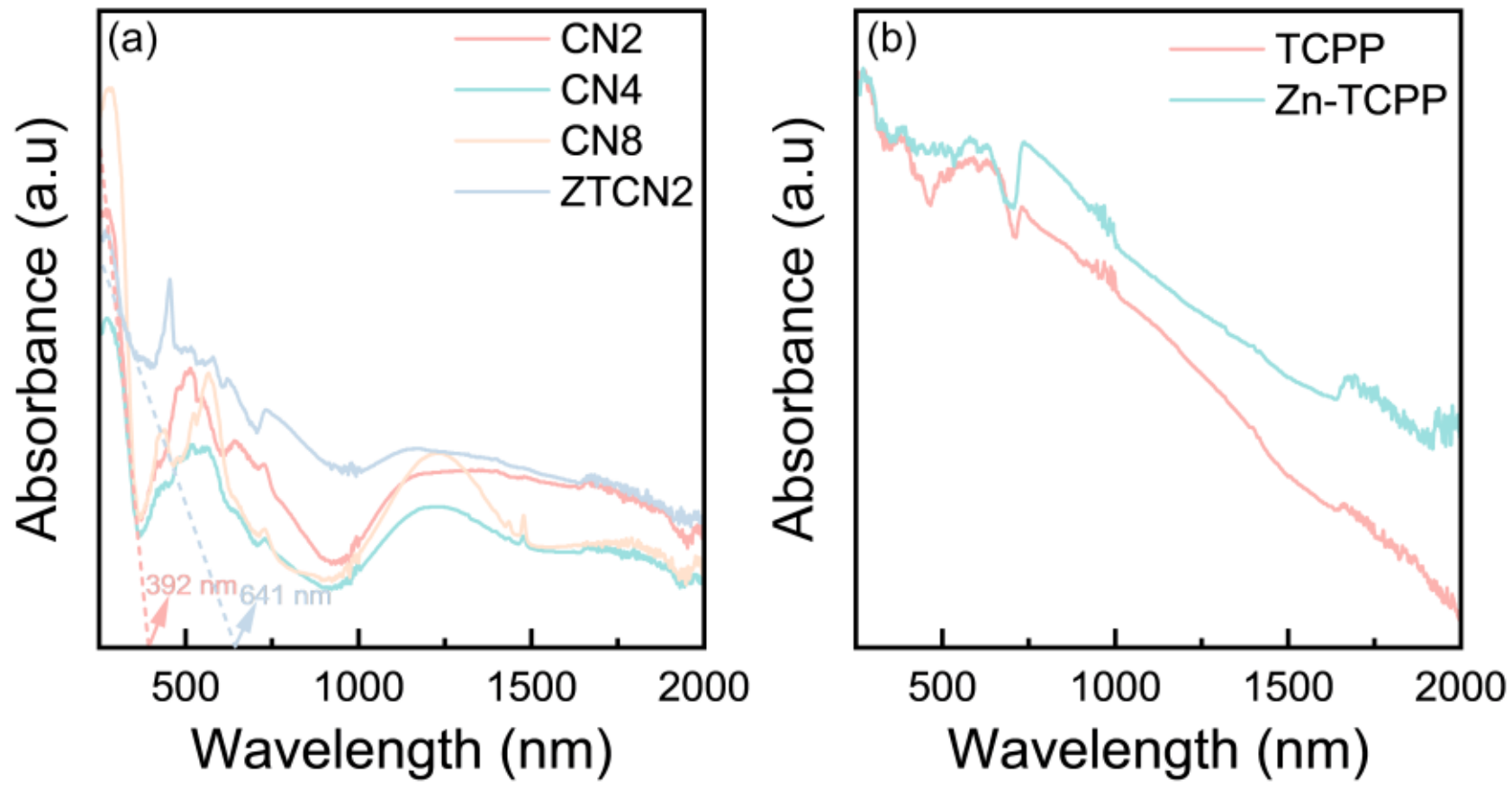

Figure S17. (a) UV-vis results of CN2, CN4, CN8 and ZTCN2. **(b)** UV-vis results of TCPP and Zn-TCPP.

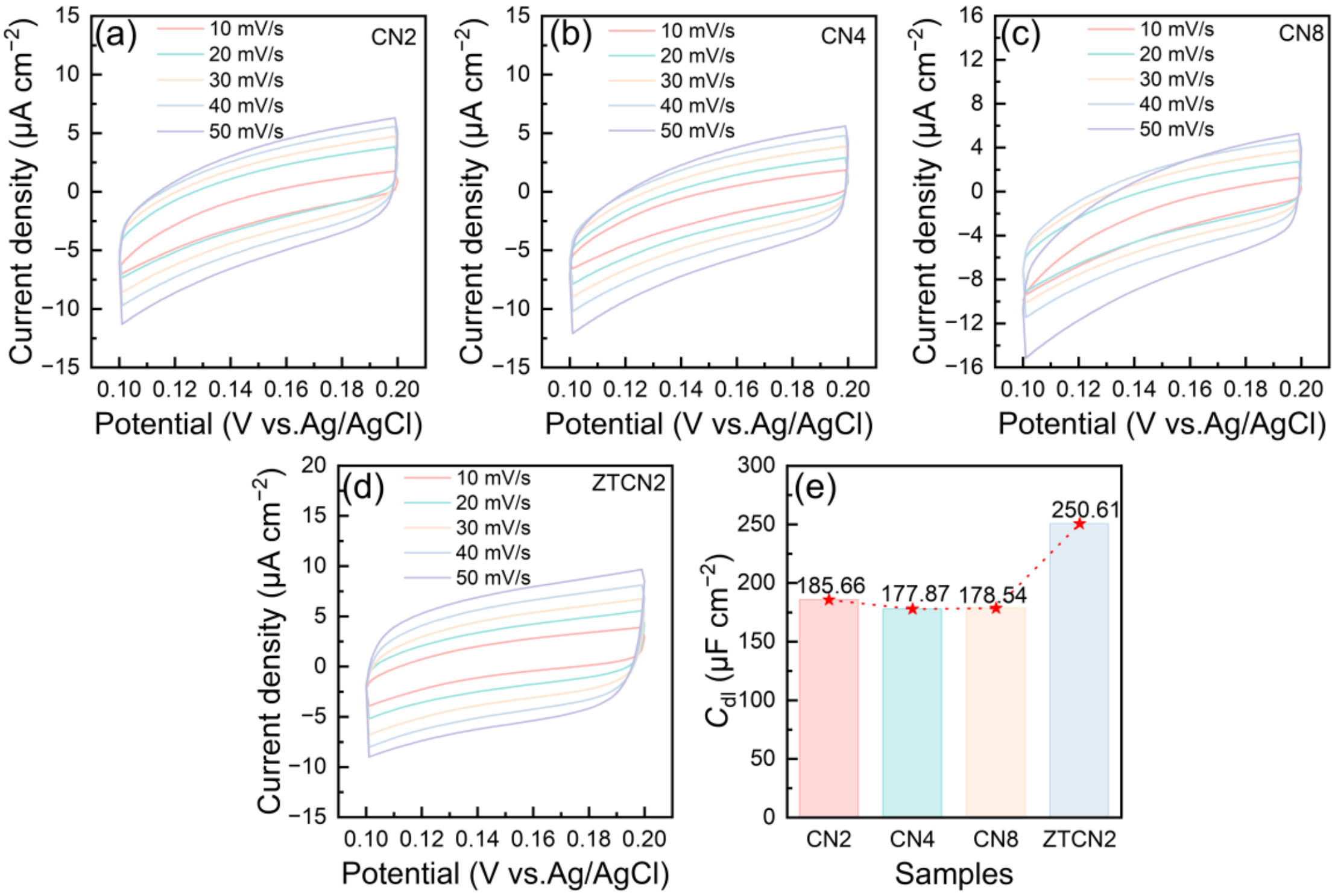

Figure S18. (a-d) CV and **(e)** comparison capacitor results of CN2, CN4, CN8 and ZTCN2 tested at different scan rates.

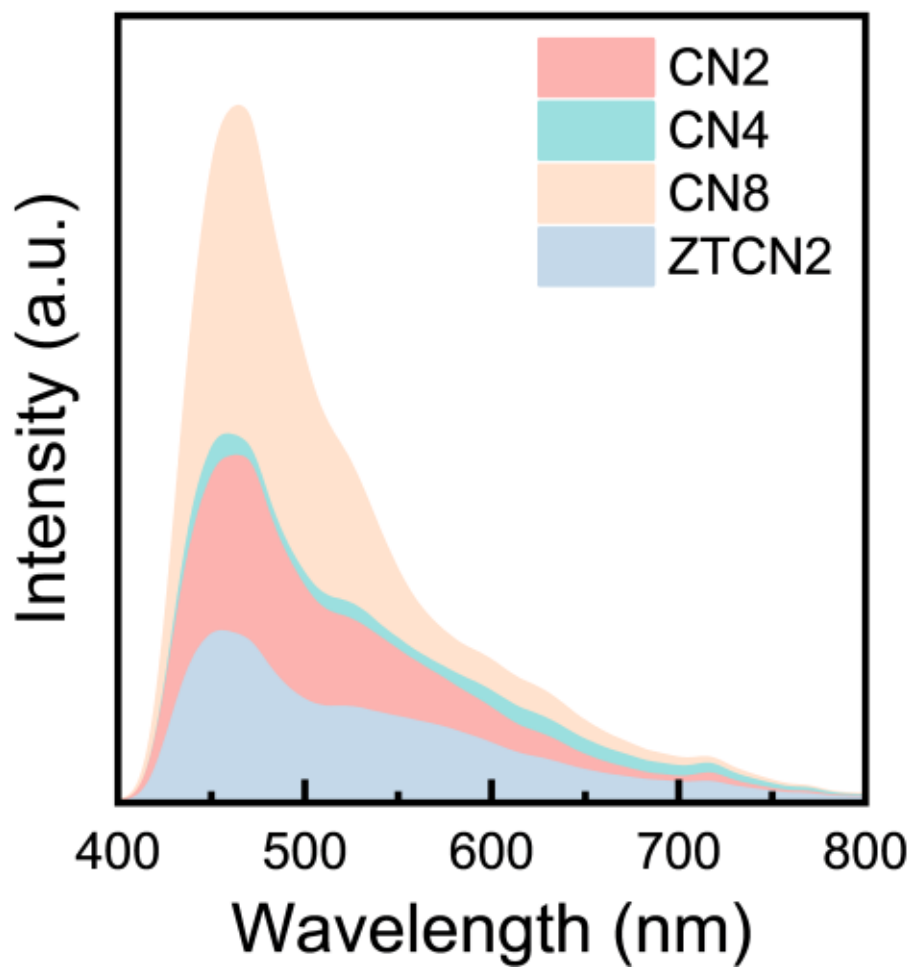

Figure S19. The PL results of CN2, CN4, CN8 and ZTCN2.

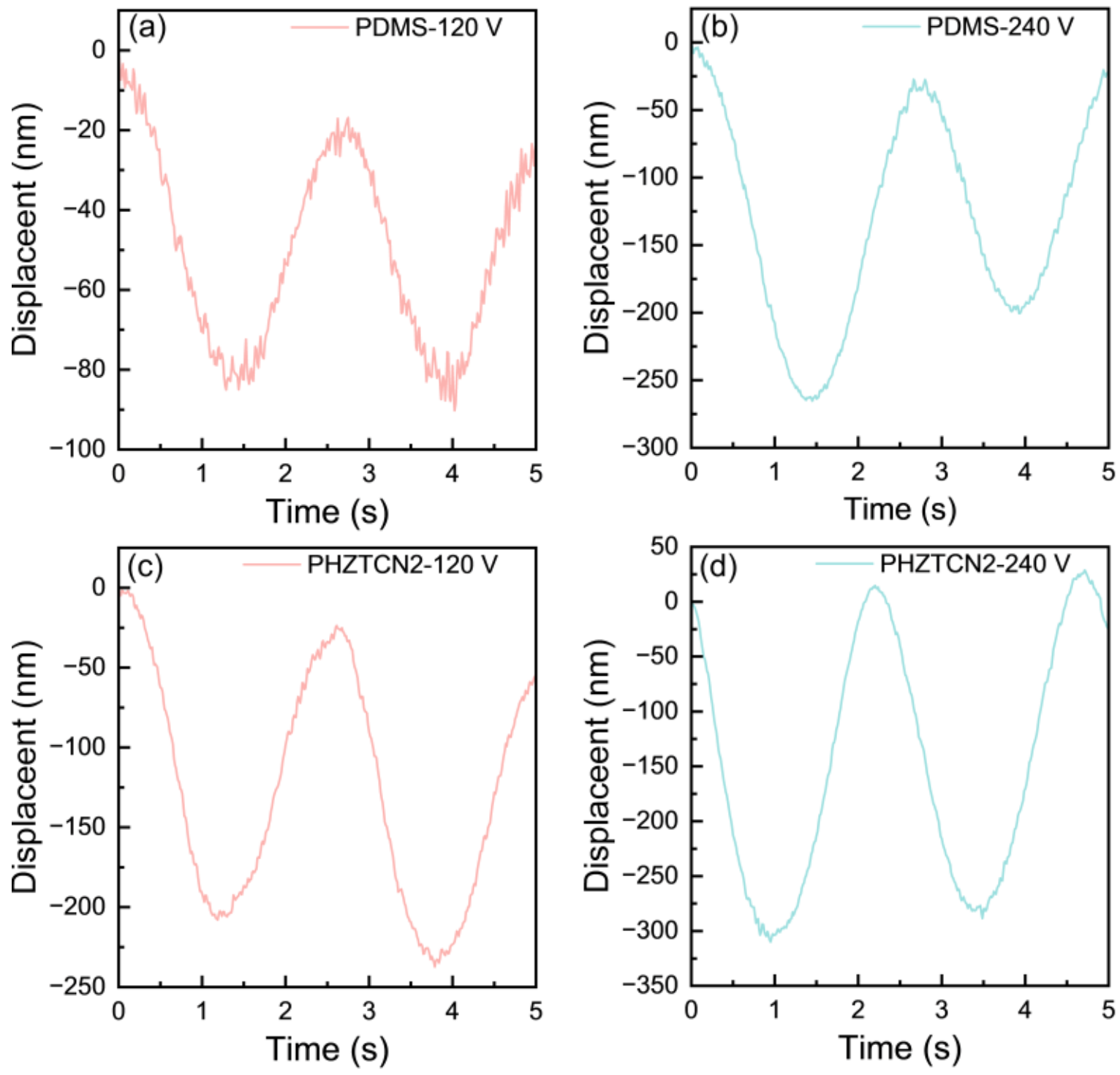

Figure S20. The deformation displacement results of **(a-b)** PDMS surfaces and **(c-d)** PHZTCN2 under different driving voltages.

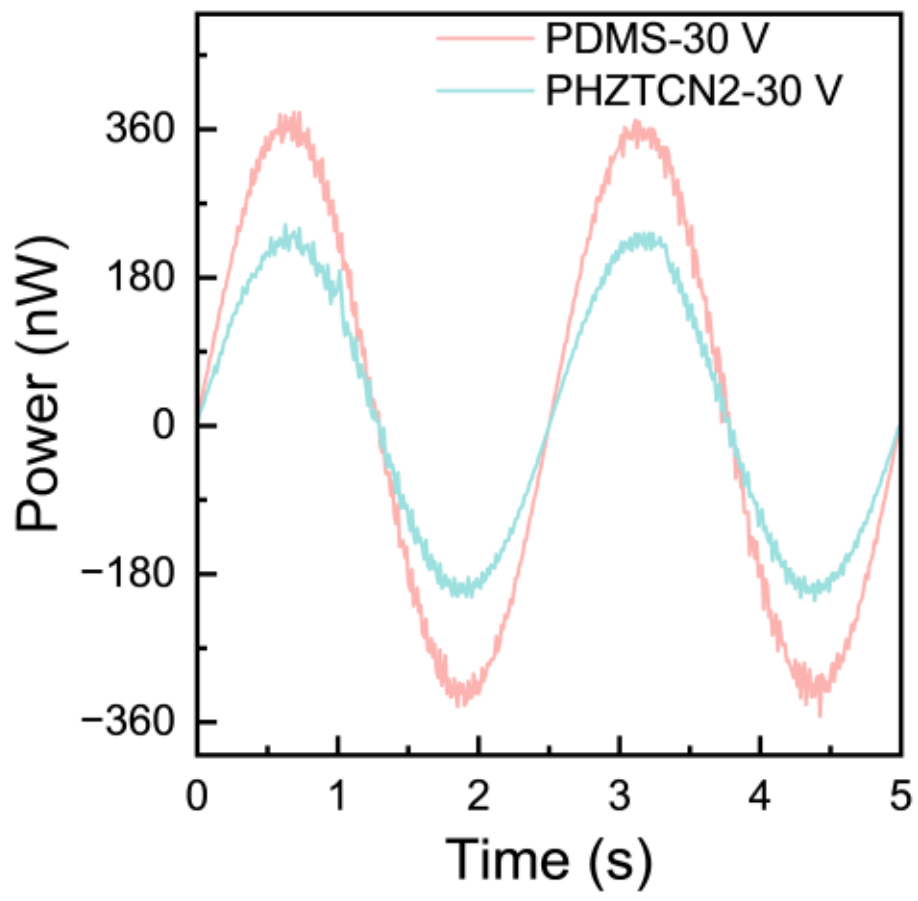

Figure S21. Instantaneous electrical power response of PDMS and PHZTCN2 under cyclic actuation at 30 V.

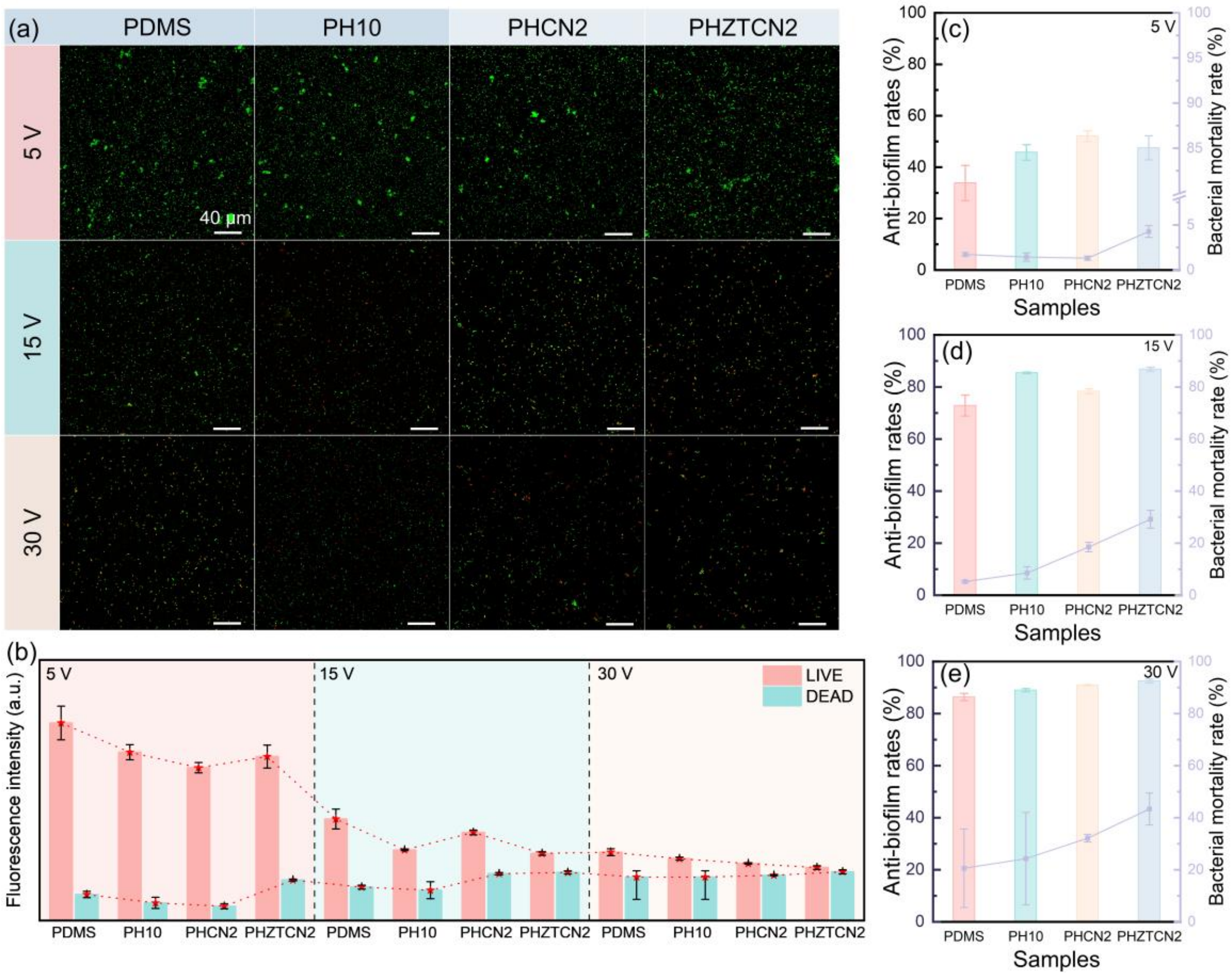

Figure S22. (a) CLSM results of PDMS, PH10, PHCN2 and PHZTCN2 under different voltages. **(b)** Fluorescence statistical results of CLSM. **(c-e)** Anti-biofilm rates and bacterial mortality rate of different surfaces under different voltages.

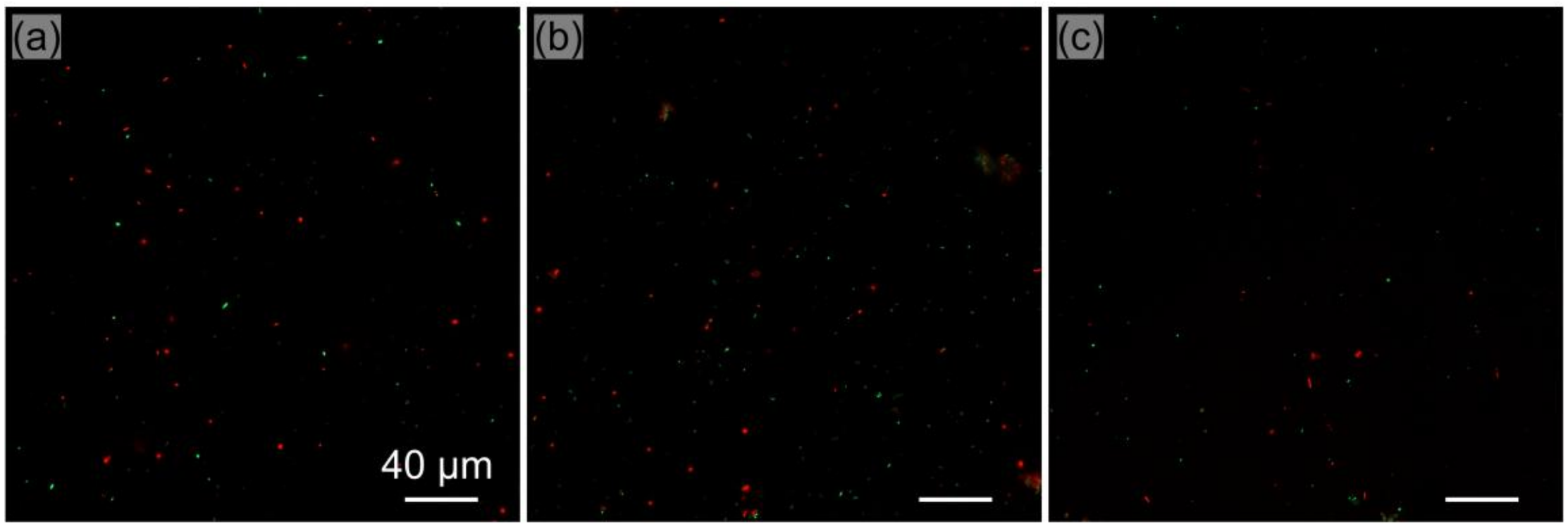

Figure S23. The CLSM results of PHZTCN2 surface after continuously working for **(a)** 1 d, **(b)** 5 d, and **(c)** 10 d.

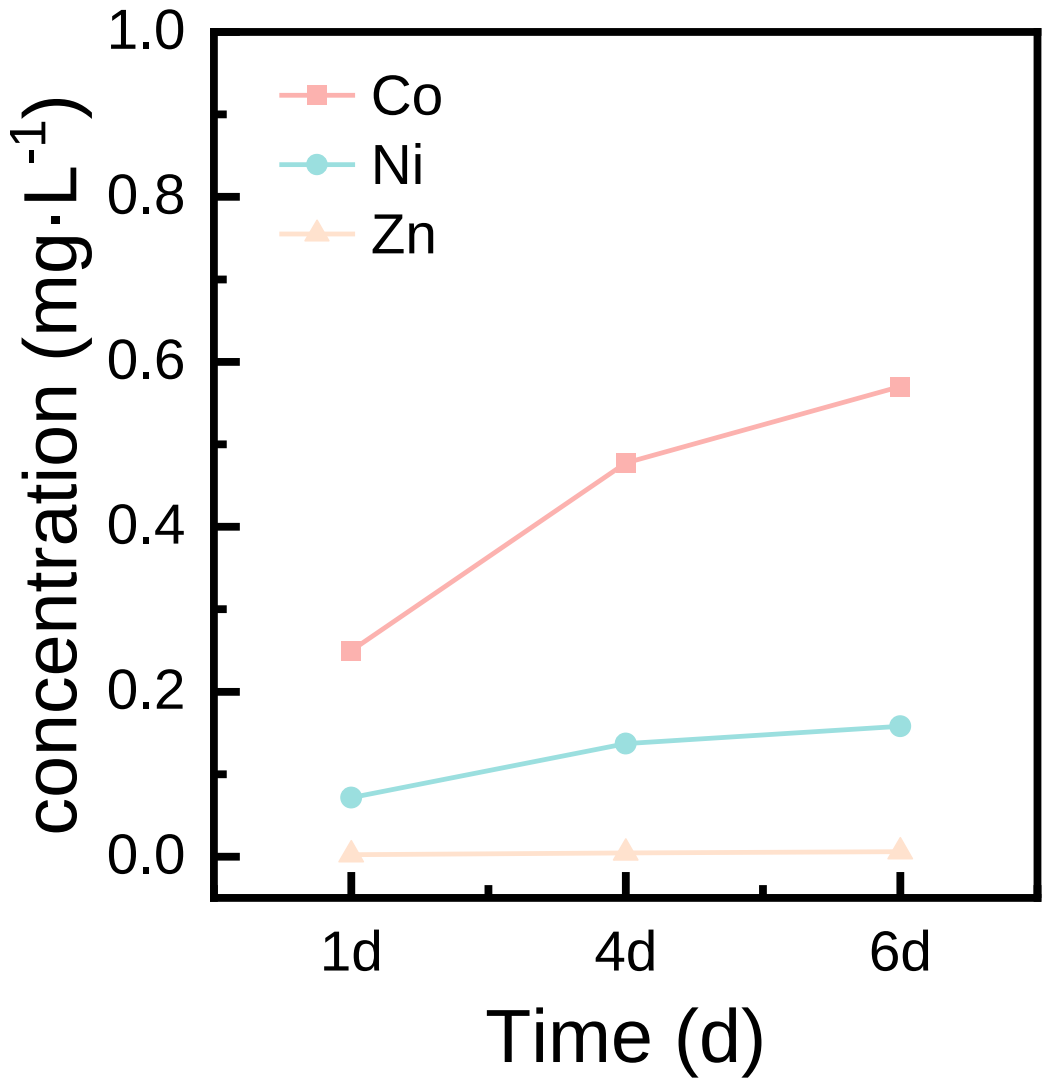

Figure S24. ICP results of PHZTCN2 after being soaked in seawater for 6 days.

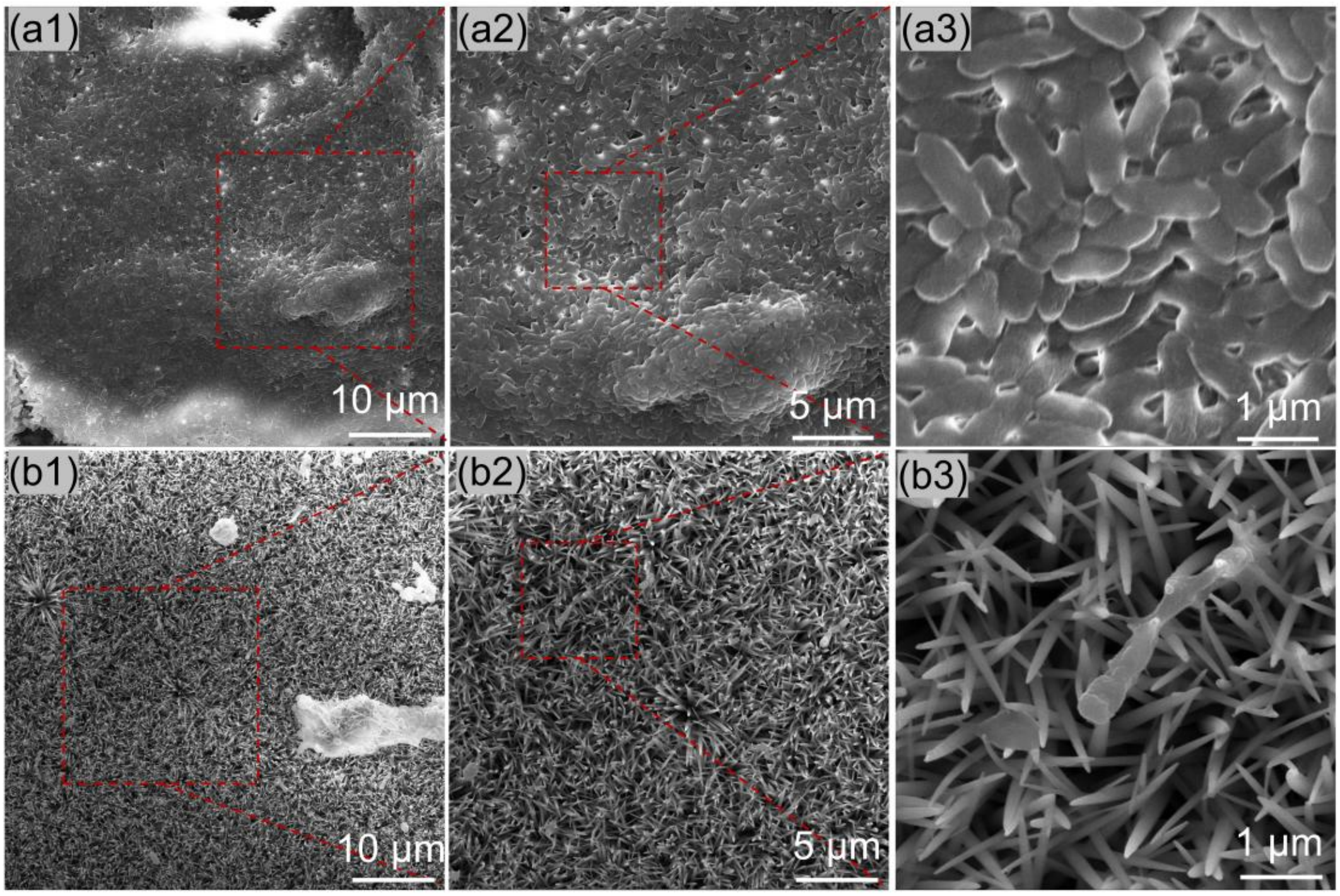

Figure S25. The SEM results of **(a)** pure Ti under dark static conditions and **(b)** PHZTCN2 under light exposure after being immersed in seawater containing *P. aeruginosa* for 1 d.

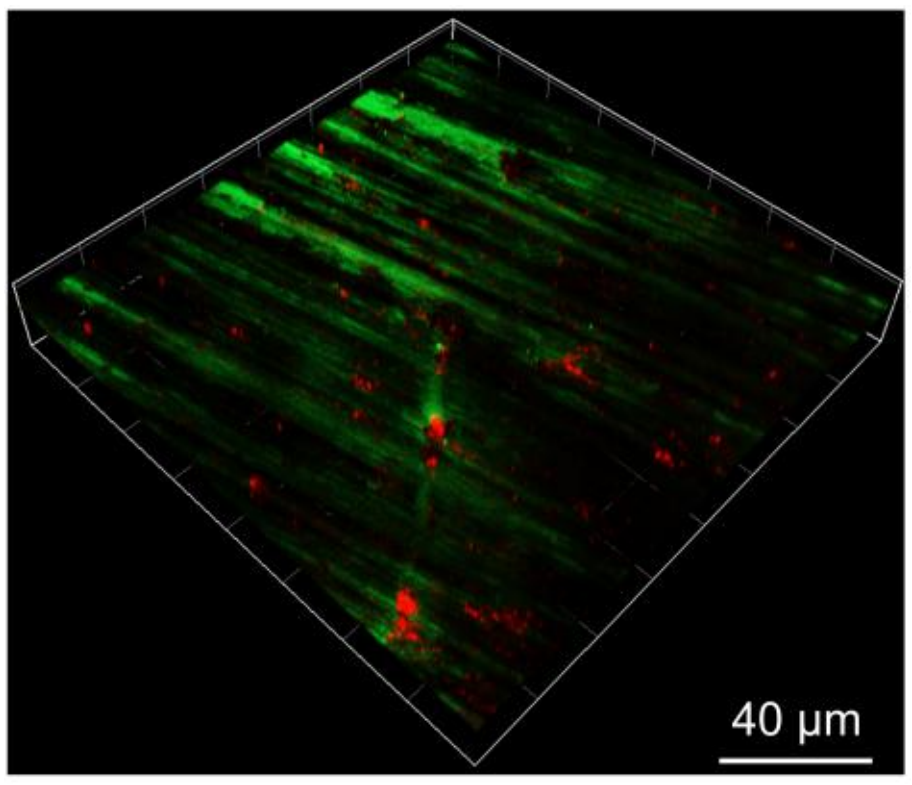

Figure S26. The CLSM results of PHZTCN2 surface after being immersed in seawater containing *P. aeruginosa* for 1 d under static, dark conditions.

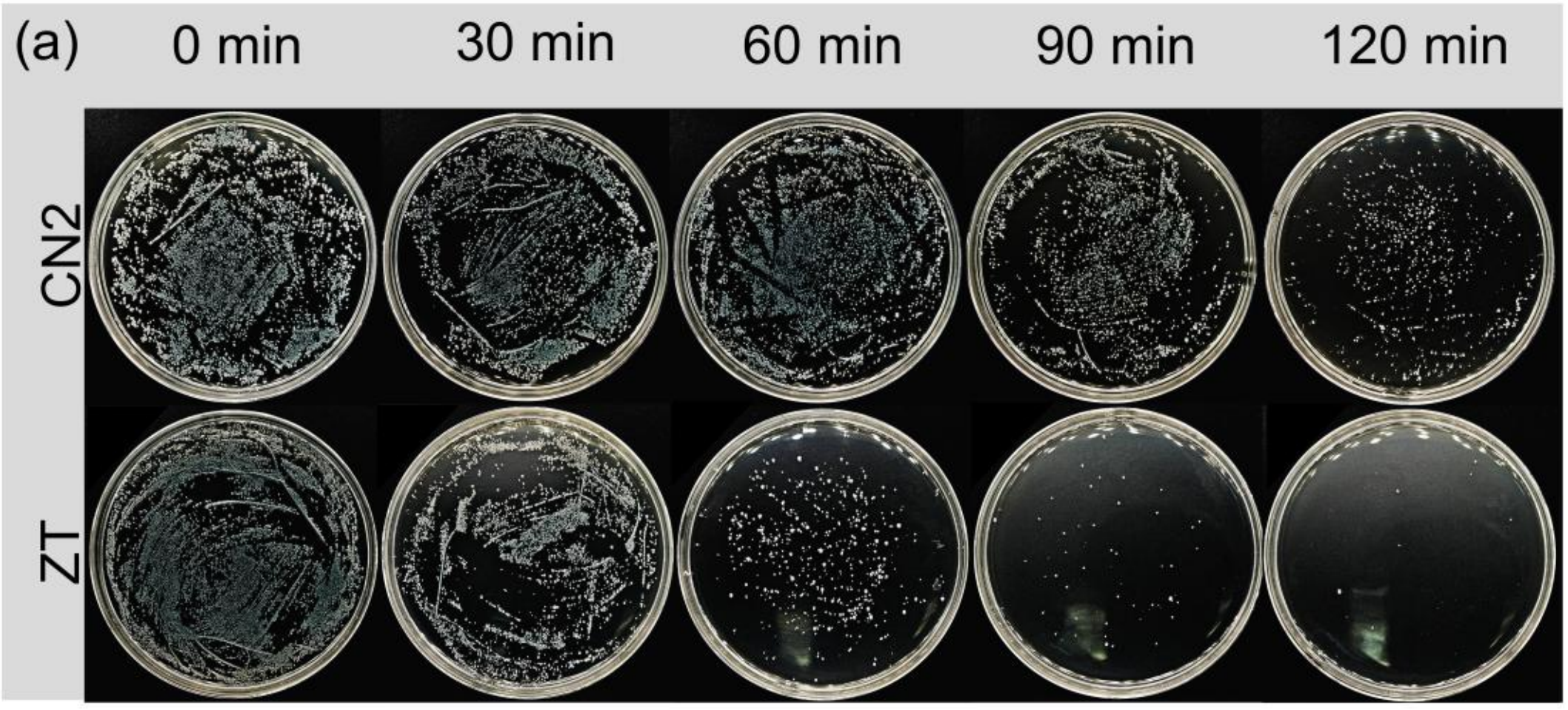

Figure S27. The plate coating results of CN2 and ZT after light-responsive free bacteria clearance within 2 h.

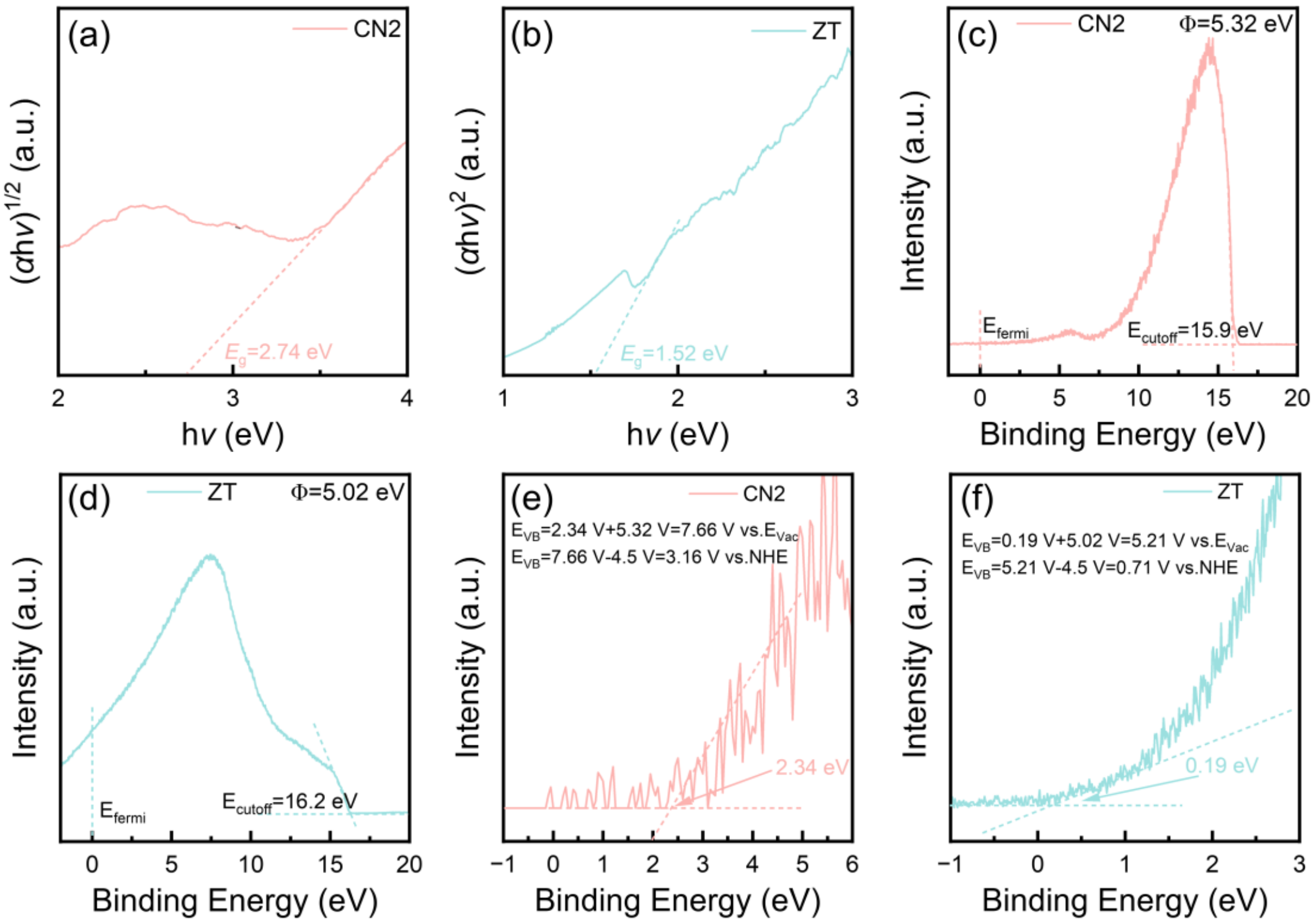

Figure S28. (a-b) Bandgap results of CN2 and ZT transformed by UV-vis. **(c-d)** Work function and **(e-f)** valence band spectrum results of CN2 and ZT tested by UPS.

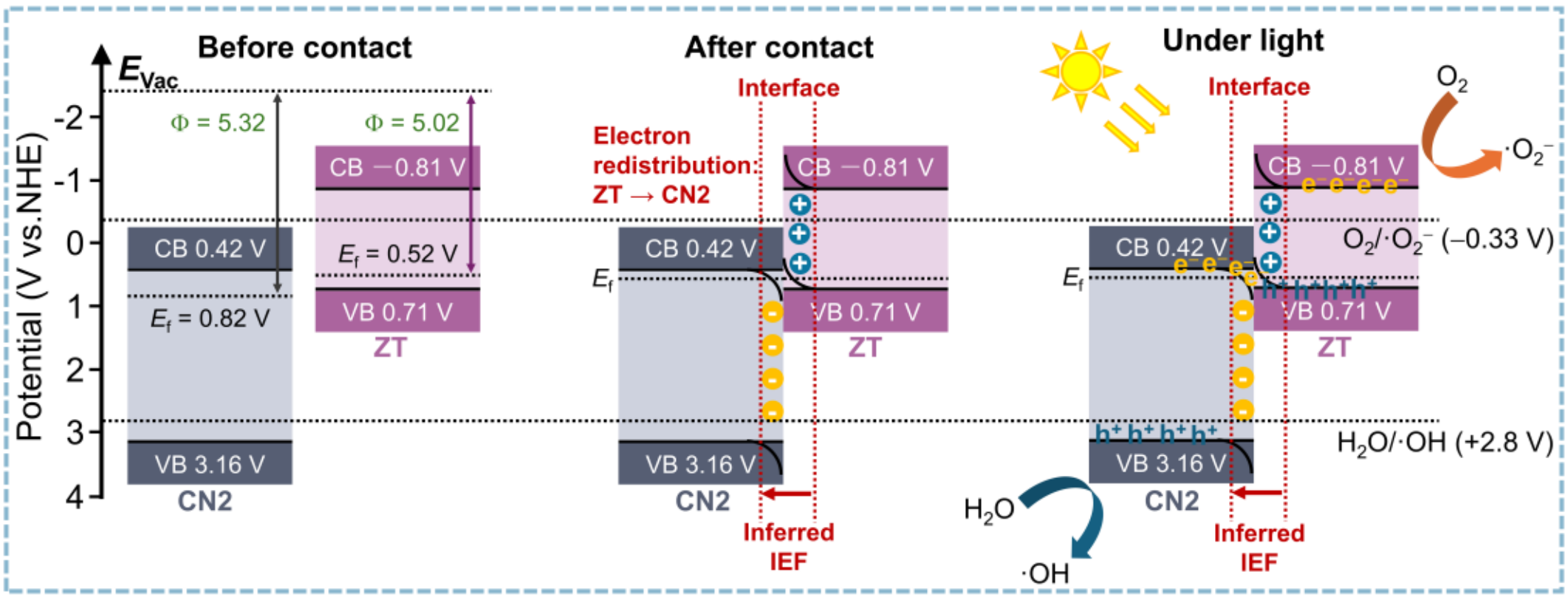

Figure S29. Proposed S-scheme charge-transfer pathway in the ZTCN2 heterostructure before contact, after contact, and under illumination. The built-in electric field direction is inferred from the work-function difference and Fermi-level equilibration.

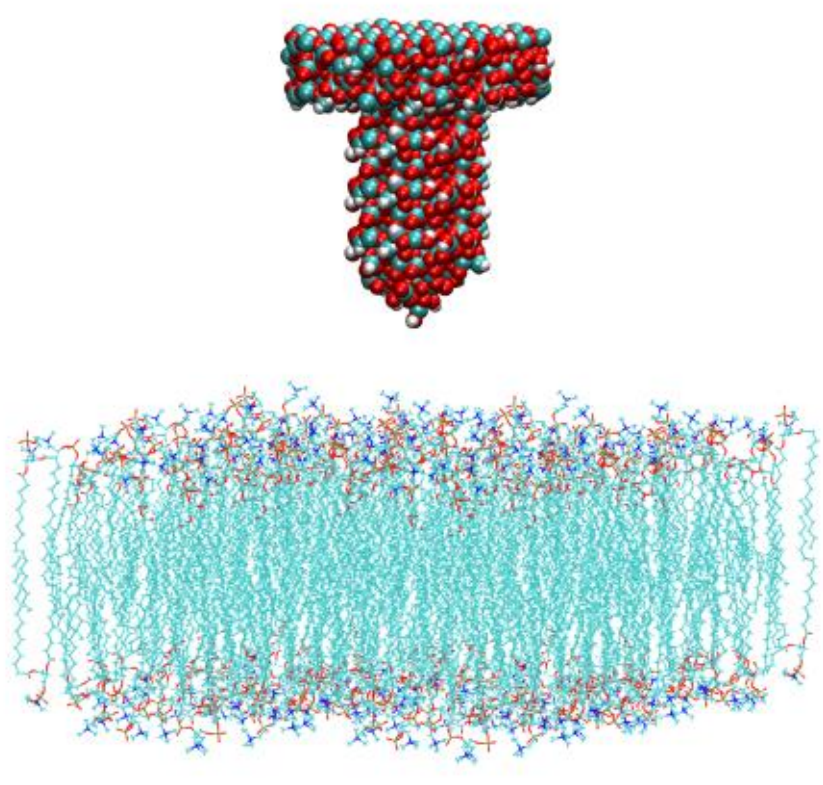

Figure S30. The modeling of tip-like structures and bacterial membranes.

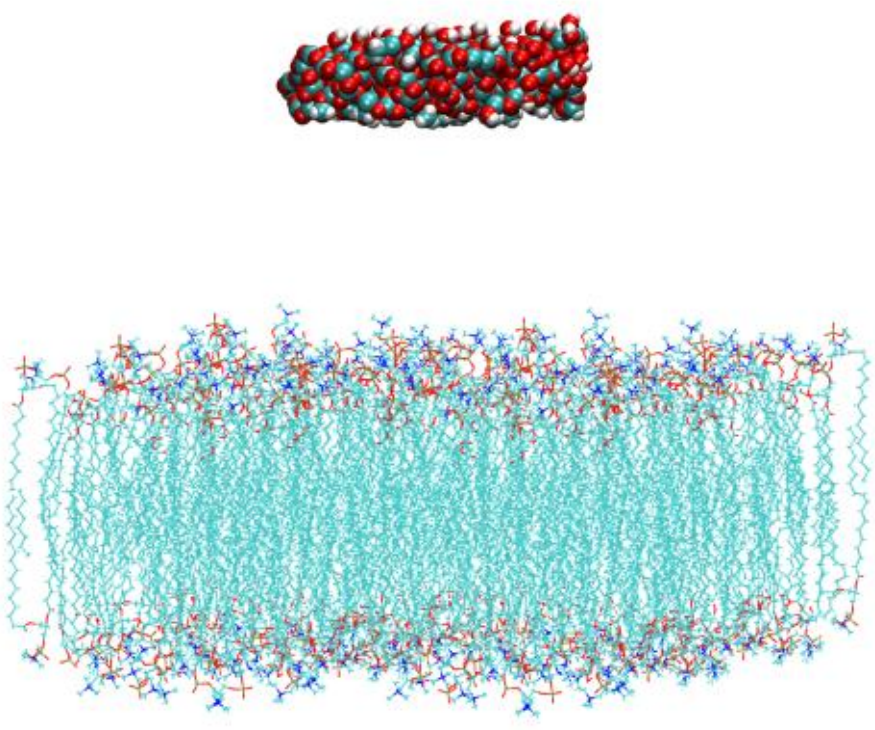

Figure S31. The modeling of planar structures and bacterial membranes.

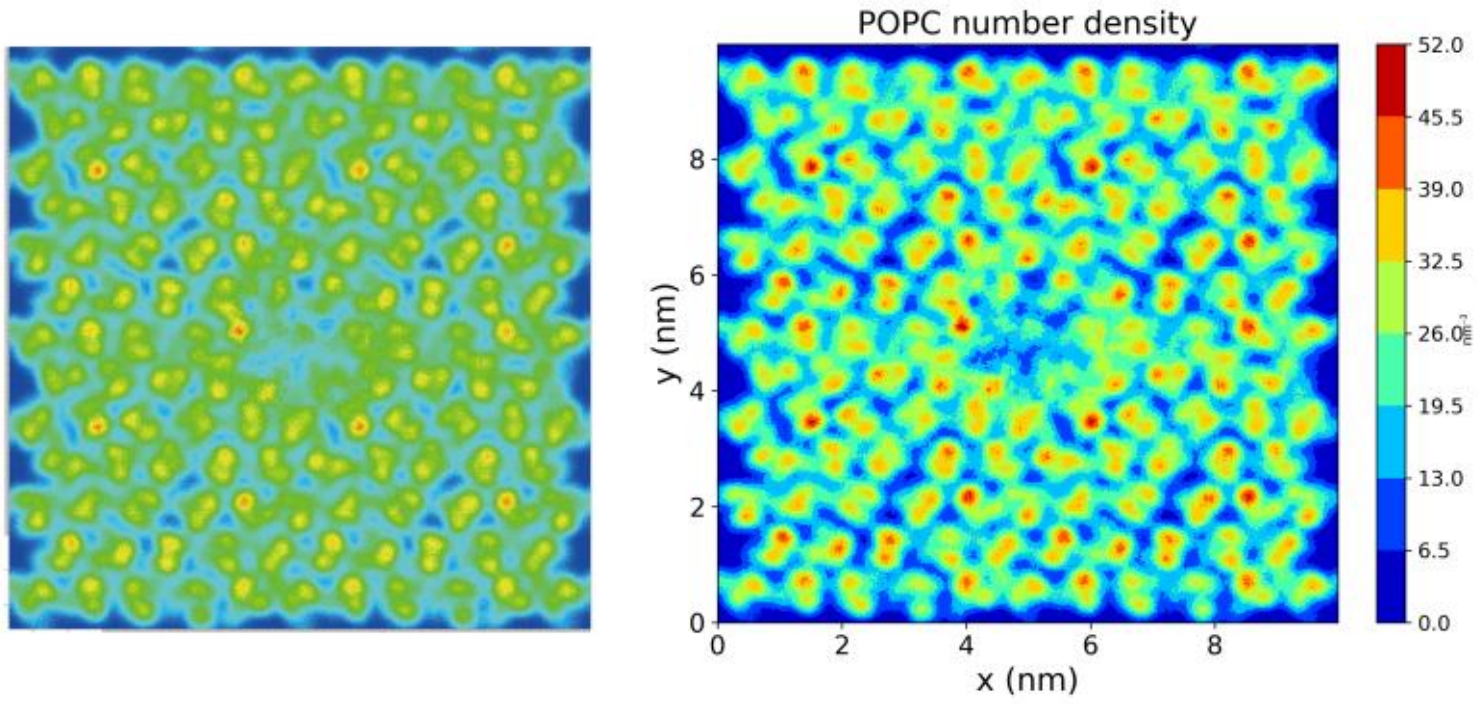

Figure S32. The membrane density results from MD simulations before and after perforation of the tip-like structure.

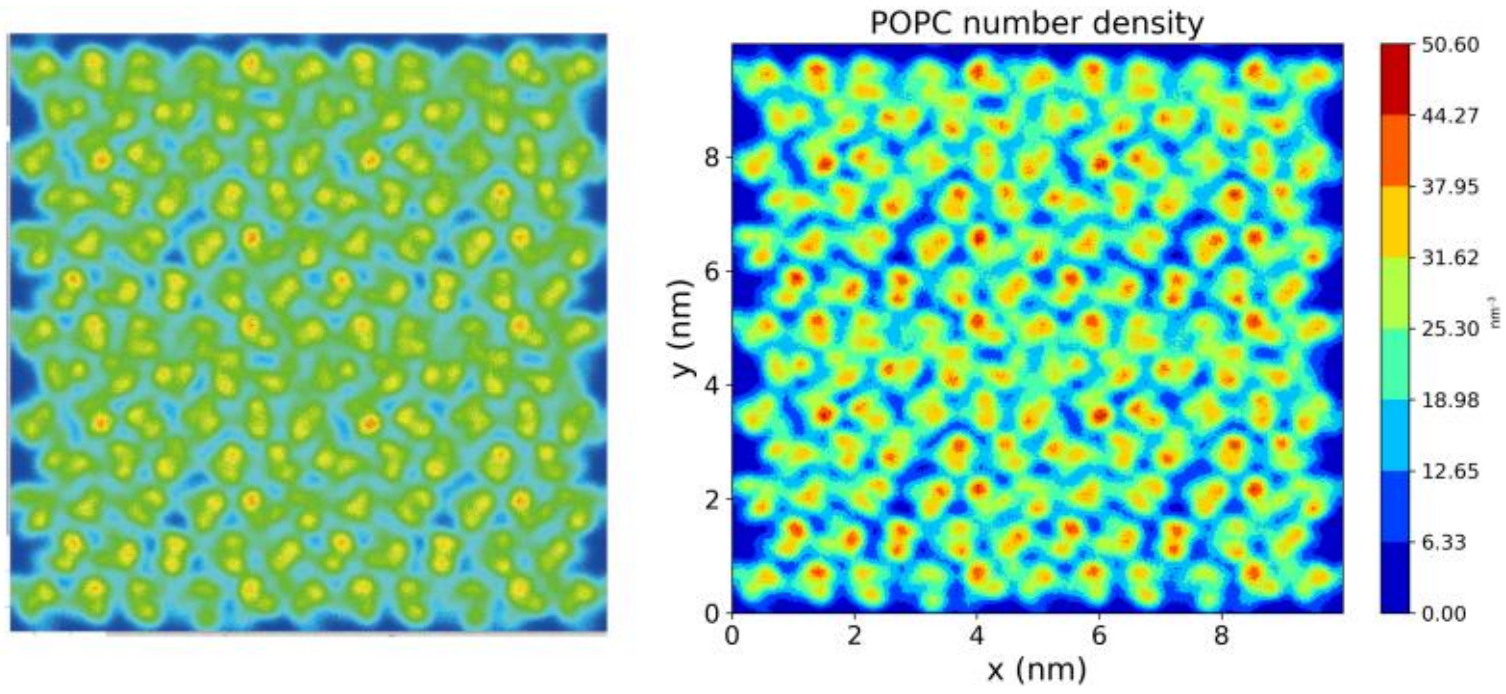

Figure S33. The membrane density results from MD simulations before and after perforation of the planar structure.

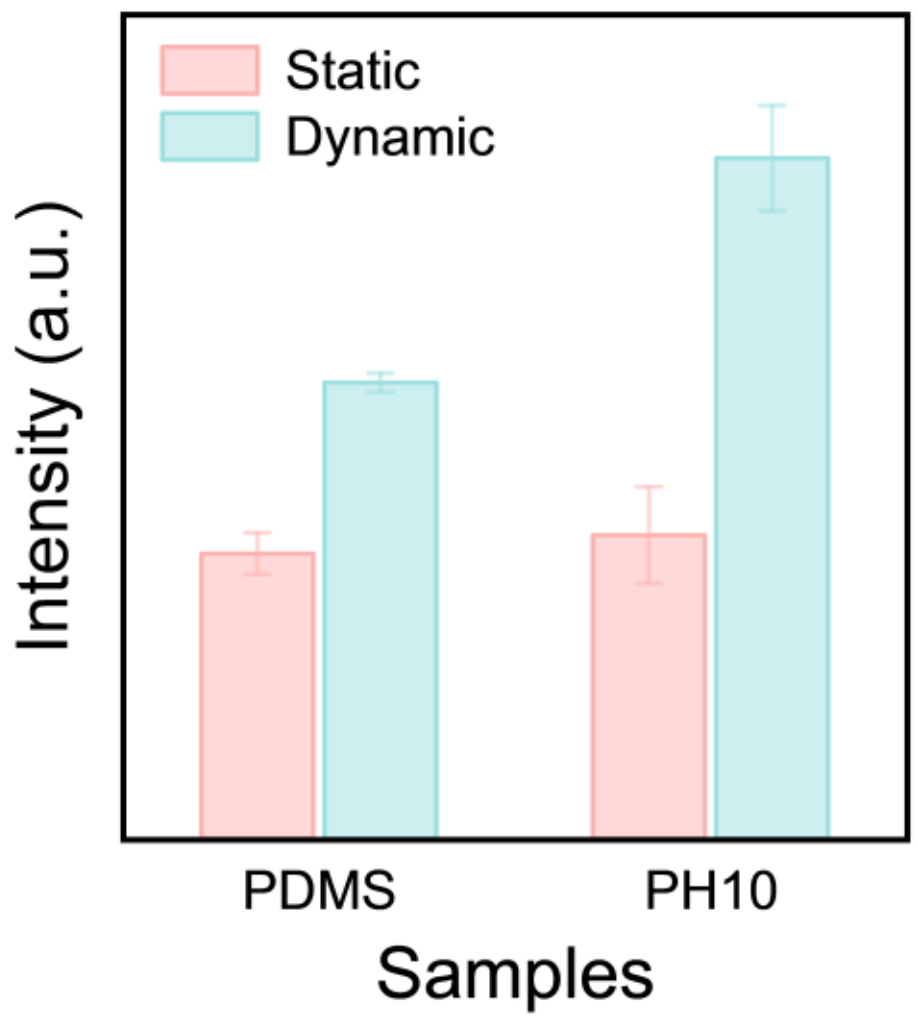

Figure S34. The membrane permeability of surface bacteria on PDMS and PH10 surfaces under static and dynamic conditions.

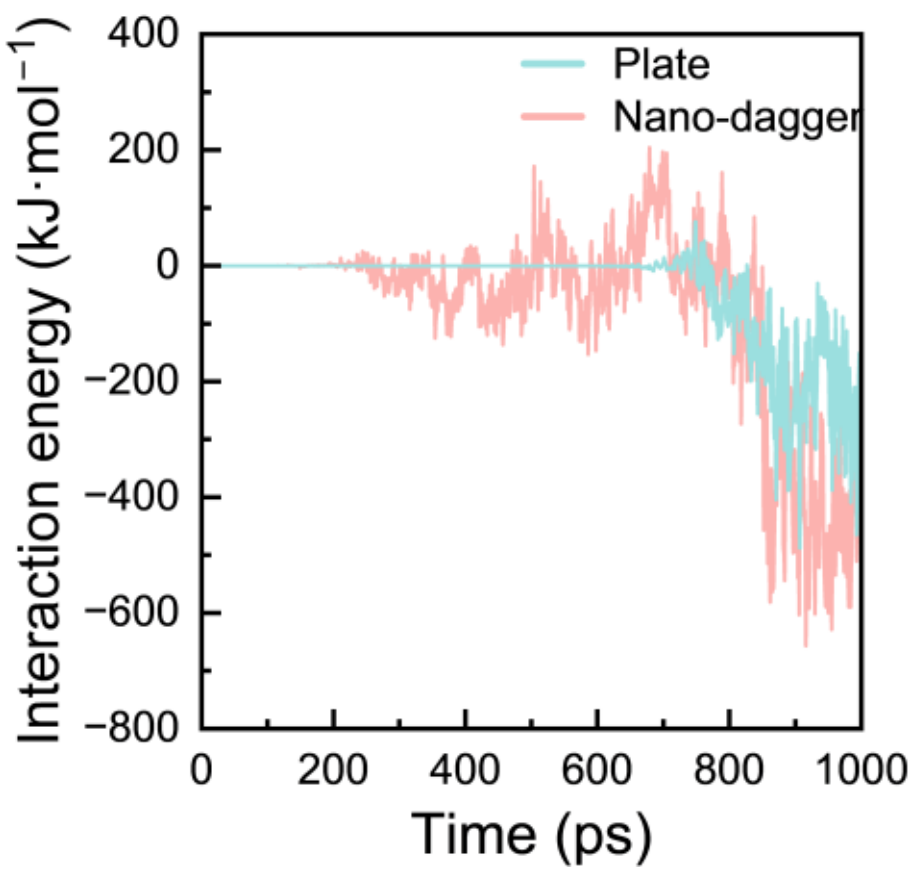

Figure S35. The interaction energy between planar and spike-like structures obtained from MD simulations and bacterial membranes related to electrostatic effects.

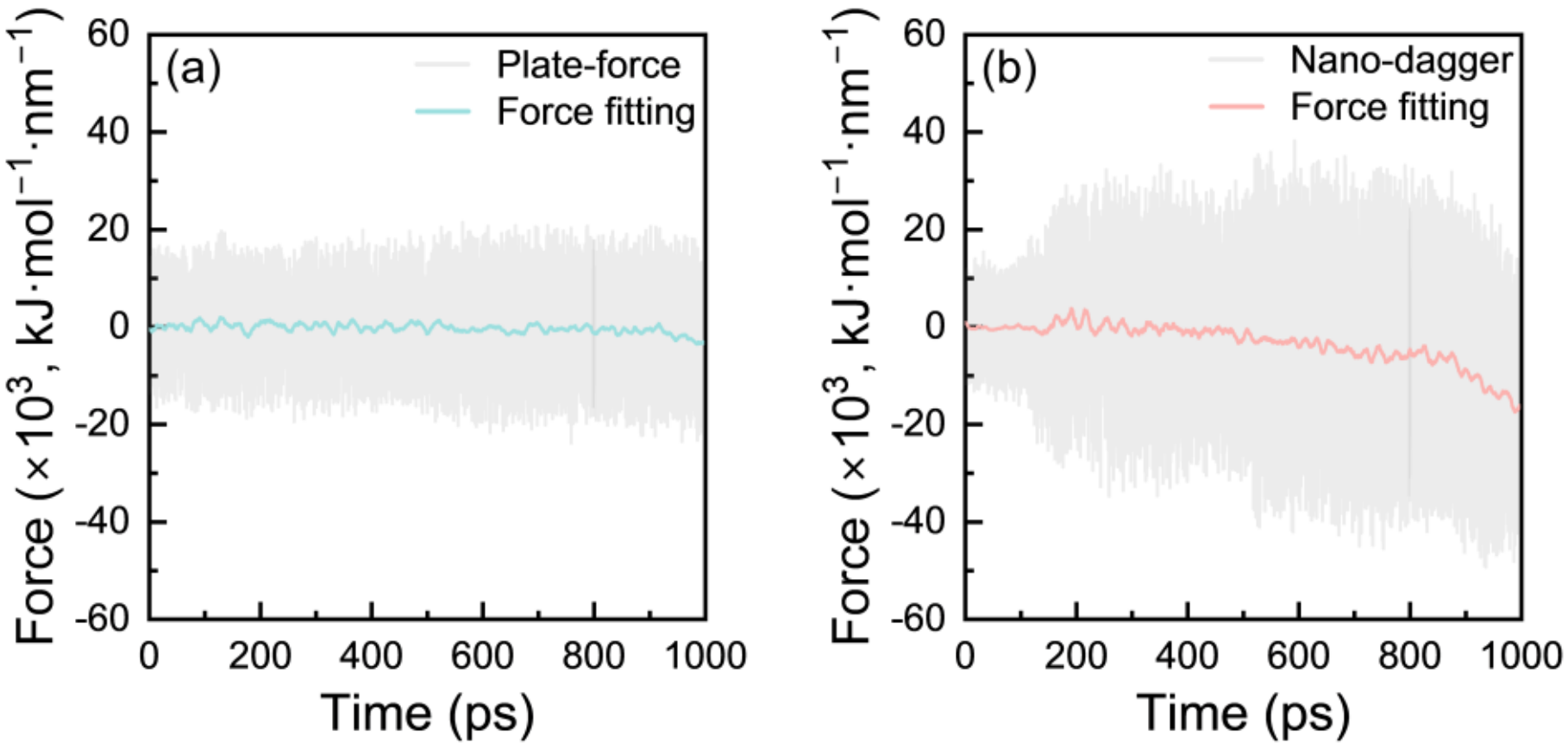

Figure S36. The force curve graphs of the **(a)** planar and **(b)** spike-like structures obtained from MD simulations interacting with bacterial membranes.

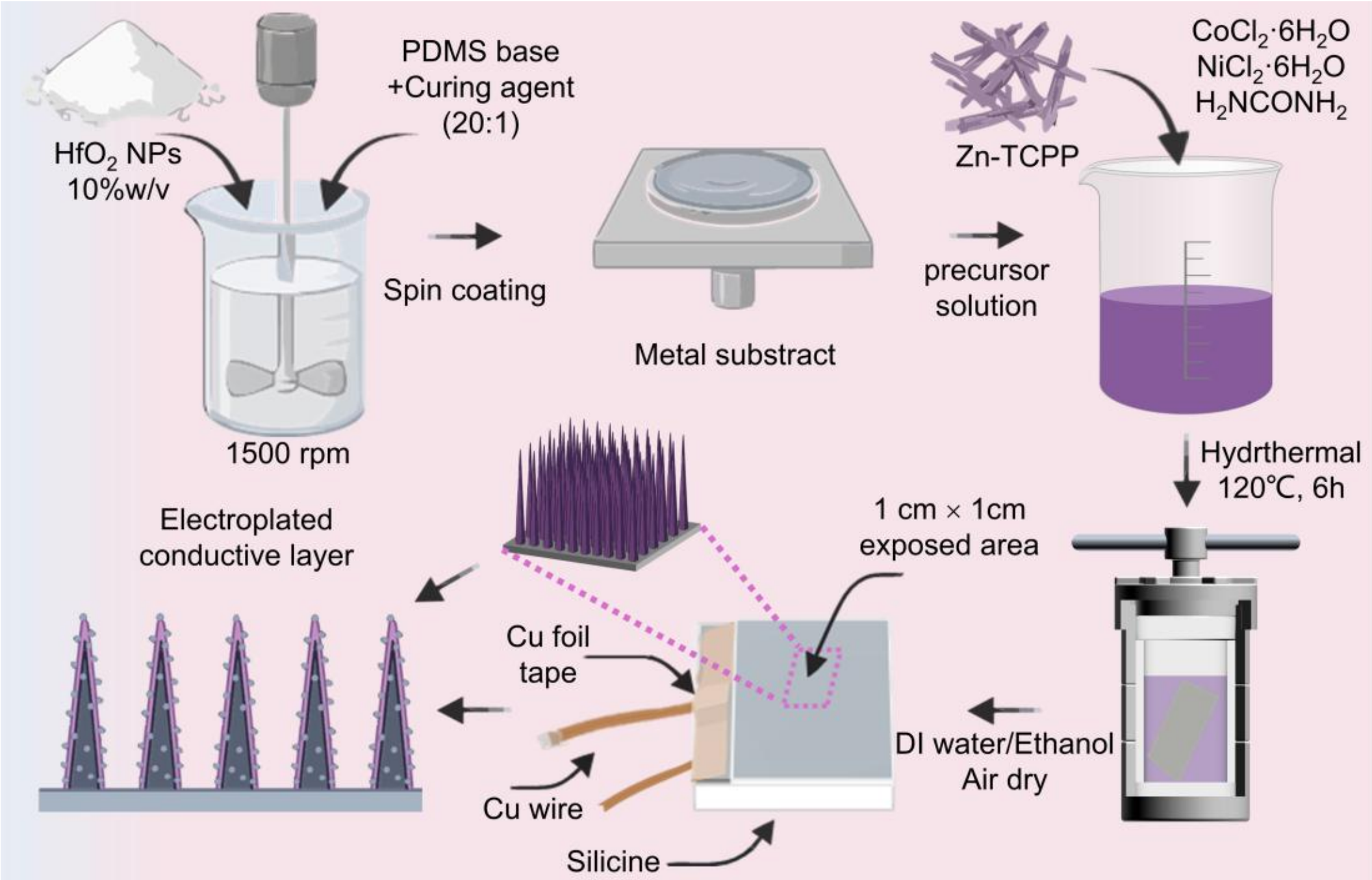

Figure S37. Schematic diagram of the fabrication process of the dynamic light-responsive nano-dagger array surface.

Table S1. Comparative summary of representative antibiofilm interfaces, including bacterial strains, inoculum concentrations and light/electrical inputs.

Material	Bacterial strain	Inoculum concentration (CFU)	Light input	Electrical input	Reference
mAPt	*S. aureus*/*S. typhimurium*	10^6	Yes	NR	[10]
+TNV	*E. coli*/*S. aureus*	10^7	NR	Yes	[11]
PDMS	*E. coli*	10^8	NR	Yes	[12]
VMSNT	MRSA	10^7	Yes	NR	[13]
Ir@Co_3O_4 (S)	MRSA	10^8	NR	NR	[14]
PEGDMA	*E. coli*	10^7	NR	NR	[15]
ZnO NW	*E. coli*/*S. aureus*	10^7	NR	NR	[16]
D@ZIF/PDA/NP	*S. aureus*/ *P. aeruginosa*	10^7	NR	NR	[17]
NanoSi	*P. aeruginosa*	10^8	NR	NR	[18]
PHZTCN2	*P. aeruginosa*	10^9	Yes	Yes	This work

Methicillin-resistant *S. aureus* (MRSA)

Not Reported (NR)

Table S2. Summary of the molecular dynamics simulation systems, including simulation-box dimensions, total atom numbers, and simulation durations.

System	Surface model	Simulation box dimensions	Total atom number	Simulation time
Planar ZTCN2-lipid-bilayer system	Planar model	100 × 100 × 180 Å^3	159073	1 ns
Nano-dagger ZTCN2-lipid-bilayer system	Nano-dagger model	100 × 100 × 180 Å^3	162111	1 ns

Reference

1. J. Jing, J. Yang, Z. Zhang, Y. Zhu, "Supramolecular Zinc Porphyrin Photocatalyst with Strong Reduction Ability and Robust Built-In Electric Field for Highly Efficient Hydrogen Production," Adv. Energy Mater. (2021), 11(29), 2101392. https://doi.org/10.1002/aenm.202101392.
2. L. Martínez, R. Andrade, E. G. Birgin, J. M. Martínez, "PACKMOL: A Package for Building Initial Configurations for Molecular Dynamics Simulations," J. Comput. Chem. (2009), 30(13), 2157. https://doi.org/10.1002/jcc.21224.
3. D. Van Der Spoel, E. Lindahl, B. Hess, et al., "GROMACS: Fast, Flexible, and Free," J. Comput. Chem. (2005), 26(16), 1701. https://doi.org/10.1002/jcc.20291.
4. J. Huang, S. Rauscher, G. Nawrocki, et al., "CHARMM36m: An Improved Force Field for Folded and Intrinsically Disordered Proteins," Nat. Methods. (2017), 14(1), 71. https://doi.org/10.1038/nmeth.4067.
5. M. Parrinello, A. Rahman, "Polymorphic Transitions in Single Crystals: A New Molecular Dynamics Method," J. Appl. Phys. (1981), 52(12), 7182. https://doi.org/10.1063/1.328693.
6. T. Darden, D. York, L. Pedersen, "Particle Mesh Ewald: An N Log (N) Method for Ewald Sums in Large Systems," J. Chem. Phys. (1993), 98(12), 10089. https://doi.org/10.1063/1.464397.
7. B. Hess, "P-LINCS: A Parallel Linear Constraint Solver for Molecular Simulation," J. Chem. Theory Comput. (2008), 4(1), 116. https://doi.org/10.1021/ct700200b.
8. A. Stukowski, "Visualization and Analysis of Atomistic Simulation Data with OVITO–the Open Visualization Tool," Modell. Simul. Mater. Sci. Eng. (2010), 18(1), 015012. https://doi.org/10.1088/0965-0393/18/1/015012.
9. W. Humphrey, A. Dalke, K. Schulten, "VMD: Visual Molecular Dynamics," J. Mol. Graphics. (1996), 14(1), 33. https://doi.org/10.1016/0263-7855(96)00018-5.
10. S. Liu, R. Shu, H. Jia, et al., "Antibody-level Bacteria Grabbing by "Mechanic Invasion" of Bioinspired Hedgehog Artificial Mesoporous Nanostructure for Hierarchical Dynamic Identification and Light-Response Sterilization," Adv. Mater. (2025), 37(15), 2416906. https://doi.org/https://doi.org/10.1002/adma.202416906.
11. G. Wang, K. Tang, W. Jiang, et al., "Quantifiable Relationship Between Antibacterial Efficacy and Electro–Mechanical Intervention on Nanowire Arrays," Adv. Mater. (2023), 35(19), 2212315. https://doi.org/https://doi.org/10.1002/adma.202212315.
12. P. Shivapooja, Q. Wang, B. Orihuela, et al., "Bioinspired Surfaces with Dynamic Topography for Active Control of Biofouling," Adv. Mater. (2013), 25(10), 1430. https://doi.org/10.1002/adma.201203374.
13. H. Zhang, D. Li, H. Ren, et al., "A Bioinspired Virus-Like Mechano–Bactericidal Nanomotor for Ocular Multidrug-Resistant Bacterial Infection Treatment," Adv. Mater. (2025), 37(8), 2408221. https://doi.org/https://doi.org/10.1002/adma.202408221.
14. S. Xiao, L. Xie, Y. Gao, et al., "Artificial Phages with Biocatalytic Spikes for Synergistically Eradicating Antibiotic-Resistant Biofilms," Adv. Mater. (2024), 36(32), 2404411. https://doi.org/10.1002/adma.202404411.
15. H. Jang, G. Choi, M. Kang, et al., "Dynamically Actuating Nanospike Composites As A Bioinspired Antibiofilm Material," Compos. Sci. Technol. (2022), 220, 109267. https://doi.org/10.1016/j.compscitech.2022.109267.
16. H.-K. Kim, S. E. Ganbaatar, R. Lopez Reyes, Y.-S. Cho, H.-H. Park, "Hydrothermally Synthesized ZnO Nanowire Surfaces: A Versatile Strategies for Antibacterial Action," Chem. Eng. J. (2025), 525, 170236. https://doi.org/10.1016/j.cej.2025.170236.
17. Z. Liu, Y. Yi, S. Wang, et al., "Bio-Inspired Self-Adaptive Nanocomposite Array: From Non-antibiotic Antibacterial Actions to Cell Proliferation," ACS Nano. (2022), 16(10), 16549.

https://doi.org/10.1021/acsnano.2c05980.
18. A. Valiei, N. Lin, J.-F. Bryche, et al., "Hydrophilic Mechano-Bactericidal Nanopillars Require External Forces to Rapidly Kill Bacteria," Nano Lett. (2020), 20(8), 5720. https://doi.org/10.1021/acs.nanolett.0c01343.
